# Integrated Multi-Omics Analysis Reveals Glycosylation Involving 2-O-β-D-Glucopyranosyl-L-Ascorbic Acid Biosynthesis in *Lycium barbarum*

**DOI:** 10.3390/ijms26041558

**Published:** 2025-02-12

**Authors:** Jiabin Huang, Haotian Wu, Ranran Gao, Lan Wu, Mengyue Wang, Yang Chu, Yuhua Shi, Li Xiang, Qinggang Yin

**Affiliations:** State Key Laboratory for Quality Ensurance and Sustainable Use of Dao-di Herbs, Artemisinin Research Center, Institute of Chinese Materia Medica, China Academy of Chinese Medical Sciences, Beijing 100700, China

**Keywords:** vitamin C, wolfberry, metabolomic, transcriptomic, carotenoids, glycosyltransferase, β-glucosidase

## Abstract

L-ascorbic acid (vitamin C, AA) is widely present in plants, but humans lack the ability to synthesize it independently. As a potent reducing agent, AA is susceptible to oxidation, making the enhancement of its stability crucial. 2-O-β-D-glucopyranosyl-L-ascorbic acid (AA-2βG) is a stable natural derivative of AA with glycosylation, initially discovered in the fruits of *Lycium barbarum*. Understanding the biosynthesis of AA-2βG is crucial for enhancing its production in *L. barbarum*. While the established biosynthesis pathway of AA constitutes the upstream of AA-2βG biosynthesis, the conclusive step of β-glycosylation remains unclear. We identified a *L. barbarum* cultivar by UPLC, ZN01, with a high content of AA-2βG, and compared its leaves, immature fruits, and mature fruits to a normal AA-2βG content *L. barbarum* cultivar for metabolomic and transcriptomic analysis. The RNA-seq and RT-qPCR analysis revealed that the expression levels of genes involved in the AA biosynthesis pathway did not consistently correlate with AA-2βG content, suggesting that the final glycosylation step may be a key determinant of AA-2βG accumulation. Subsequently, utilizing phylogenetic and co-expression analysis, we identified ten UDP-glycosyltransferases (UGTs) and three β-glucosidases (BGLUs) which may be involved in the crucial step of the conversion from AA to AA-2βG, and the UGTs’ activities were predicted through molecular docking. Lastly, we speculated that the presence of the glycosylation process of AA might have a crucial role in maintaining AA homeostasis in *L. barbarum*, and deliberated on potential correlations between AA, carotenoids, and anthocyanins. Our integrated multi-omics analysis provides valuable insights into AA-2βG biosynthesis in *L. barbarum*, identifying thirteen candidate genes and highlighting the complex interplay between AA, carotenoids, and anthocyanins. These findings have implications for improving AA-2βG content in *L. barbarum*.

## 1. Introduction

*Lycium barbarum* L. is a deciduous shrub with red and yellow fruits, also known as wolfberry and goji berry, which belongs to the Solanaceae family. The fruit of a wolfberry is rich in polysaccharides, betaine, phenolics, carotenoids, and vitamins [1]. Due to the medicinal and nutritional benefits of its fruit, it is widely cultivated in Asia and Europe [2,3]. L-ascorbic acid (vitamin C, AA) is one of the most abundant primary metabolites in plants, and it demonstrates antioxidant capacity and an ability to terminate oxidative chain reactions [4], but humans and several other groups of animals have lost their ability to synthesize AA and often need additional intake [5]. However, the instability of the C2 hydroxyl group in AA makes it highly susceptible to oxidative degradation under oxygen, heat, or pH changes [6]. Previous studies have utilized various methods to enhance the stability of AA, resulting in the synthesis of ascorbates and glycosylated derivatives [7]. 2-O-β-D-glucopyranosyl-L-ascorbic acid (AA-2βG) is a natural AA glycosylated derivative that exists in *L. barbarum* [8] which exhibits enhanced chemical stability [9], and possesses antioxidant [10,11] and modulating gut microbiota [12].

The biosynthesis pathway of AA constitutes the upstream of AA-2βG synthesis. Previous studies have demonstrated four pathways for the biosynthesis of AA in plants: the D-mannose/L-galactose pathway [13], the D-galacturonate pathway [14], the L-gulose pathway [15], and the myo-inositol pathway [16]. The D-mannose/L-galactose pathway, commonly known as the Smirnoff–Wheeler pathway, is the primary route for AA biosynthesis. The 10 genes encoding enzymes in the pathway convert α-D-glucopyranose to AA: nicotinate/nicotinamide mononucleotide adenyltransferase (NMNAT), phosphoglucose isomerase (PGI), phosphomannose isomerase (PMI), phosphomannomutase (PMM), GDP-D-mannose pyrophosphorylase (GMP), GDP-D-mannose 3′,5′-epimerase (GME), GDP-L-galactose phosphorylase (GGP), L-galactose-1-phosphate phosphatase (GPP), L-galactose dehydrogenase (L-GalDH), and L-galactono-1,4-lactone dehydrogenase (L-GalLDH) [17]. Related studies also reported myo-inositol oxygenase (MIOX) in the myo-inositol pathway and D-galacturonate reductase (GalUR) in the D-galacturonate pathway [14,16]. The recycling pathways of AA involve multiple genes. Monodehydroascorbate reductase (MDHAR) and dehydroascorbate reductase (DHAR) promote AA regeneration, while ascorbate peroxidases (APX) and ascorbate oxidase (AO) catalyze the oxidation of AA, and glutathione reductase (GR) facilitates the AA-GSH cycle by regenerating reduced glutathione (GSH) from its oxidized form (GSSG) to assist the AA regeneration process [18].

*Lycium ruthenicum* Murr., also known as black wolfberry, accumulates abundant anthocyanins in fruit. Some studies showed that no AA-2βG and no total carotenoid content was detected in the fruits of *L. ruthenicum*, while no total anthocyanin content was detected in the fruits of *L. barbarum* [19,20]. This reflects two patterns of color accumulation in wolfberry fruits: anthocyanin accumulation pattern and carotenoid accumulation pattern. We hypothesize that the metabolic flux changes associated with these two patterns may also be related to other metabolic pathways, leading to differences in the content of AA-2βG. *L. barbarum* fruits contain a rich amount of zeaxanthin (a type of β-carotene), primarily existing in the esterified form as zeaxanthin dipalmitate [21]. The biosynthesis pathway of carotenoids is conserved in plants, with approximately 12 classes of genes playing crucial roles. Phytoene synthase (PSY), known as the primary rate-limiting step in carotenoid biosynthesis, is followed by phytoene desaturases (PDS), ζ-carotene isomerase (ZISO), ζ-carotene desaturases (ZDS), and carotenoid isomerase (CRTISO), which convert uncolored phytoene to colored lycopene. Lycopene β-cyclase (LCYb) and lycopene ε-cyclase (LCYe) can cyclize lycopene into β-carotene and α-carotene, respectively. β-carotene can be converted to zeaxanthin by non-heme carotene hydroxylases (BCH) and further transformed into corresponding compounds by zeaxanthin epoxidase (ZEP), which could reverse by violaxanthin de-epoxidase (VDE) to form the xanthophyll cycle. Carotenoid cleavage oxygenase (CCO), including carotenoid cleavage dioxygenase (CCD) and 9-*cis*-epoxycarotenoid dioxygenase (NCED), contribute to produce apocarotenoids and to maintain the steady-state levels of carotenoids [22,23].

Although the upstream biosynthesis pathway of AA-2βG has been clarified, the mechanism of the final glycosylation step remains unclear. The key to this glycosylation step lies in the difference in the α/β configuration of the linkage between AA and the sugar moiety. The isomer 2-O-α-D-glucopyranosyl-L-ascorbic acid (AA-2αG) of AA-2βG is commercially available, and its synthesis has been extensively reported. Cyclodextrin glycosyltransferase (CGTase), amylase, α-isomaltosyl glucosaccharide-forming enzyme (IMGase), α-glucosidase, and sucrose phosphorylase (SPase) have been used to synthesize AA-2αG [7]. Hydroquinone can be glycosylated to produce α and β configurations. Hydroquinone to α-arbutin can be synthesized by the amylosucrase (ASase) cloned from *Deinococcus geothermalis* [24]. As early as a decade ago, arbutin, the O-β-D-glucoside of hydroquinone, was demonstrated to be synthesized using RsAs (a UGT cloned from *Rauvolfia serpentina* cell suspension cultures) [25]. Most UGTs mediate β-glucosylation, but only one study has reported that overexpression of *UGT87A2* in *Arabidopsis thaliana* can increase its AA-2βG content, and there is no direct in vitro evidence to support this finding [26]. A patent reported that 5,6-O-(isopropylidene) ascorbic acid can be catalyzed by galactosidase to generate 2-O-(β-D-Galactopyranosyl) ascorbic acid [27], which also falls under the category of β-glycosylation of ascorbic acid analogs. Fortunately, cellulase-related enzymes from *Trichoderma virus*, *Aspergillus niger*, and *Trichoderma reesei* have been proved to be able to convert AA and cellobiose to AA-2βG [28]. These preliminary studies provide us with important references.

In this study, we identified seven cultivars of *L. barbarum* with differ phenotype. In order to reveal key enzymes in AA-2βG biosynthesis, two cultivars, ZN01 and ZN02, with significant differences in AA-2βG contents were selected for further non-targeted metabolomic (NGM) and RNA-seq analysis. Based on metabolomic and transcriptomic data, we constructed the biosynthesis pathway of AA in *L. barbarum* to analyze the spatiotemporal distribution differences of AA-2βG. We also speculated on the enzyme involved in the glycosylation process during the final step. Additionally, we annotated the carotenoid biosynthesis pathway in *L. barbarum* and analyzed the changes in carotenoid content and types during the transition of fruits from immature (green, G) to mature (red, R) stages. This study provides the foundation for identifying the key enzyme in the final step of AA-2βG biosynthesis in *L. barbarum* and explores the potential role of the AA-2βG precursor AA in the accumulation patterns of anthocyanins and carotenoids.

## 2. Results

### 2.1. Difference of AA-2βG Content in Cultivars of L. barbarum

**Figure 1:**
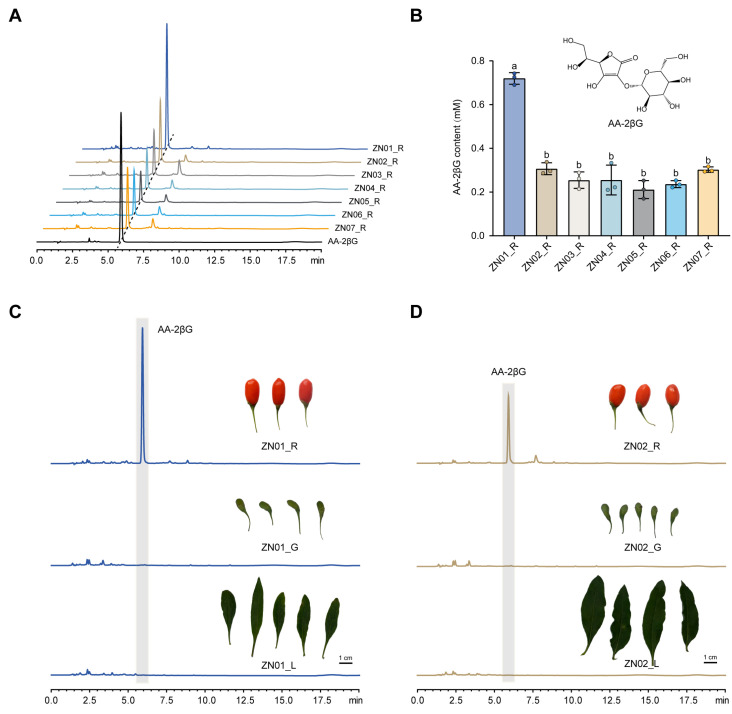
Phenotypes of different cultivars of *Lycium barbarum*. (**A**) UPLC profiles of AA-2βG in mature (red) fruits of seven different cultivars of *L*. *barbarum* with UV detection at 260 nm. (**B**) Content of AA-2βG in red fruits of seven different cultivars of *L*. *barbarum* and the structural formula of AA-2βG. The lowercase letters indicate significance levels at *p* ≤ 0.05, as determined by Tukey’s test. (**C**) UPLC profiles of AA-2βG and phenotypic maps of red fruits (ZN01_R), green fruits (ZN01_G), and leaves (ZN01_L) of ZN01. (**D**) UPLC profiles of AA-2βG and phenotypic maps of red fruits (ZN02_R), green fruits (ZN02_G), and leaves (ZN02_L) of ZN02. The error bars represented means ± SD from three replicates.

Metabolites from mature (red, R) fruits of seven different *L. barbarum* cultivars were extracted using water, and the content of AA-2βG was quantified (Figure 1A). The findings revealed that the cultivar ZN01_R (red fruits of ZN01) exhibited the highest AA-2βG content (0.72 mM) compared to the other cultivars, with a content 3.4 times higher than that of the cultivar ZN05_R, which had a low AA-2βG content (0.21 mM) level. There were no significant differences in the AA-2βG content among the other cultivars, namely ZN02_R, ZN03_R, ZN04_R, ZN05_R, ZN06_R, and ZN07_R (Figure 1A,B). Furthermore, to investigate the accumulation of AA-2βG at various growth stages of the fruits, the AA-2βG content was assessed in immature (green, G) fruits of ZN02 and ZN01 (ZN02_G and ZN01_G), with no detectable AA-2βG content observed under UPLC analysis. Additionally, an analysis of the leaves of ZN02 and ZN01 (ZN02_L and ZN01_L) also did not reveal the presence of AA-2βG (Figure 1C,D). Therefore, the differential accumulation of AA-2βG may mainly occur during the period of fruit ripening transition.

### 2.2. Temporal and Spatial Specificity of AA-2βG Biosynthesis in L. barbarum

**Figure 2:**
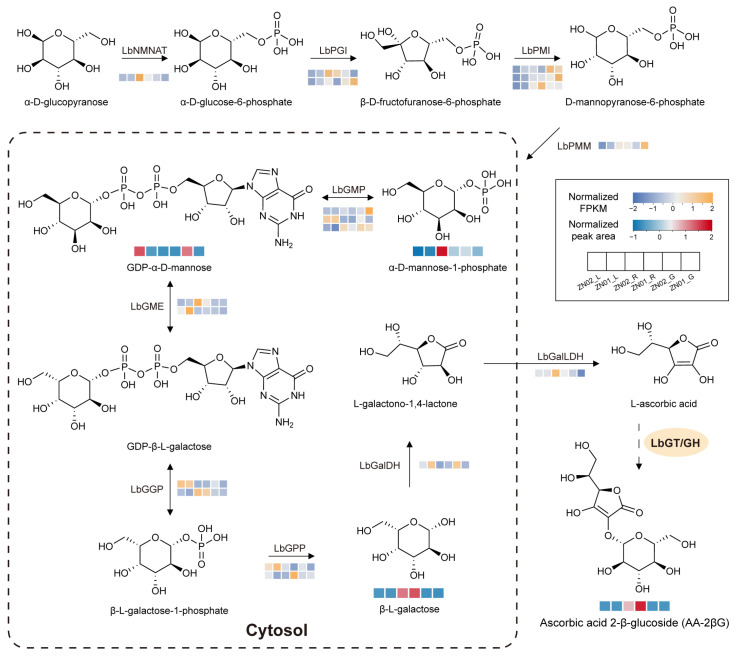
Proposed biosynthesis pathway of AA-2βG in *L*. *barbarum*. The biosynthetic pathway of AA refers to the Smirnoff–Wheeler pathway. The color scale from purple to orange of heatmaps below genes refers to the gene expression value from low to high. Heatmaps below the compounds indicate their relative quantification in different tissues, with content ranging from low (blue) to high (red). The enzyme that synthesizes AA-2βG from AA is presumed to be glycosyltransferase/glycoside hydrolase (LbGT/GH), but it has not yet been resolved, indicated here by a dashed arrow. NMNAT, nicotinate/nicotinamide mononucleotide adenyltransferase; PGI, phosphoglucose isomerase; PMI, phosphomannose isomerase; PMM, phosphomannomutase; GMP, GDP-D-mannose pyrophosphorylase; GME, GDP-D-mannose 3′,5′-epimerase; GGP, GDP-L-galactose phosphorylase; GPP, L-galactose-1-phosphate phosphatase; L-GalDH, L-galactose dehydrogenase; L-GalLDH, L-galactono-1,4-lactone dehydrogenase.

To further investigate the variations in AA-2βG content across organs and different stages of fruit growth, we conducted non-targeted metabolomic (NGM) and RNA-seq analyses on the mature fruit, immature fruit, and leaves of ZN02 and ZN01. The quality control data for RNA-seq is presented in Table S1, and the results indicate that the quality meets acceptable standards. Principal component analysis (PCA) was performed to evaluate 18 samples, comprising three biological replicates from different cultivars and tissue samples. In the transcriptome analysis, PC1 and PC2 accounted for 47.1% and 23.8% of the variance, respectively. In the metabolome analysis, PC1 and PC2 explained 31.8% and 23.8% of the variance, respectively. The three biological replicates of each sample clustered closely, while significant differences in transcriptional and metabolic profiles were observed between distinct samples (Figure S1). By referencing the Smirnoff–Wheeler pathway, we investigated the AA biosynthesis pathway in *L. barbarum* (Figure 2). In this pathway, four compounds were identified through metabolomic analysis: α-D-mannose-1-phosphate, GDP-α-D-mannose, β-L-galactose, and ascorbic acid 2-β-glucoside (AA-2βG) (Table S2). α-D-mannose-1-phosphate was notably abundant in ZN02_R and also present in ZN02_G, ZN01_R, and ZN01_G, while in lower levels in leaves. Conversely, the detection of GDP-α-D-mannose yielded unexpected results, with only minor accumulation observed in ZN02_R and ZN01_R. The accumulation pattern of β-L-galactose in the downstream pathway mirrored that of AA-2βG, being predominantly enriched in mature fruits. Consistently, the amount of AA-2βG detected in leaves and immature fruits was traced, aligning with our findings (Figure 1C,D).

We annotated 18 genes involved in the biosynthesis pathway of AA and examined their transcription levels using RNA-seq analysis (Figure 2 and Table S3). Among these genes, *LbNMNAT*, *LbPGI1*, *LbPMI1*, *LbPMI3*, *LbGME1*, *LbGGP2*, and *LbGalLDH* exhibited predominant expressions in mature fruits, with only *LbPMI3* showing higher expression in ZN01_R compared to ZN02_R. Some genes displayed high expression levels in immature fruits, such as *LbPGI2*, *LbPMI2*, *LbPMM*, *LbGMP1*, and *LbGMP3*. Notably, the expression levels of these genes were higher in ZN01_G than ZN02_G, except for *LbGMP3*, which may directly lead to differential accumulation of GDP-α-D-mannose in immature fruits. The expression patterns of *LbGMP2*, *LbGME2*, *LbGGP1*, *LbGPP1*, and *LbGalDH* in leaves did not align with the accumulation patterns of the detected metabolites. Only the elevated expression of *LbGMP2* appears to promote the enrichment of GDP-α-D-mannose. Furthermore, we identified *LbGalUR* in the D-galacturonate pathway and *LbMIOX* in the myo-inositol pathway as complementary pathways for AA biosynthesis. Specifically, *LbGalUR7*, *LbGalUR8*, and *LbGalUR9* exhibited high expression in mature fruits, while *LbMIOX1* and *LbMIOX2* were highly expressed in immature fruits, potentially playing a role in the synthesis of AA. In AA recycling pathways, *LbDHAR1* was highly expressed in immature fruits, while *LbMDHAR* showed high expression in mature fruits. *LbAPX* (1, 3, 7, and 8) and *LbAO*1 were mainly expressed in mature fruits (Figure S2 and Table S3). Among these genes, the expression pattern of *LbGalDH* and *LbGalLDH*, which are most critical for AA synthesis, was not correlated with the accumulation pattern of AA-2βG. This may indicate the significance of the final glycosylation step for the high enrichment of AA-2βG in mature fruits.

Network analysis was employed to examine the differentially expressed metabolites (DEGs) between ZN01 and ZN02. We identified the anthocyanin biosynthesis pathway (ath00942) and galactose metabolism (ath00052) within the network, while these pathways were not directly associated with each other (Figure S3 and Tables S4 and S5). Furthermore, we performed KEGG enrichment analysis on the DEGs from various samples. The analysis revealed that DEGs in the fruits and leaves of the same cultivar were enriched in fructose and mannose metabolism (lbb00051), and in carotenoid biosynthesis (lbb00906) (Figure S4A,C and Tables S6 and S8). In contrast, the genes differentially expressed between mature and immature fruits were primarily enriched in carotenoid biosynthesis (lbb00906) (Figure S4B,D and Tables S7 and S9). Notably, fructose and mannose metabolism (lbb00051) suggested a link to the biosynthesis of AA, while the carotenoid biosynthesis pathway has also captured our interest.

### 2.3. Temporal and Spatial Specificity of Carotenoid Biosynthesis in L. barbarum

**Figure 3:**
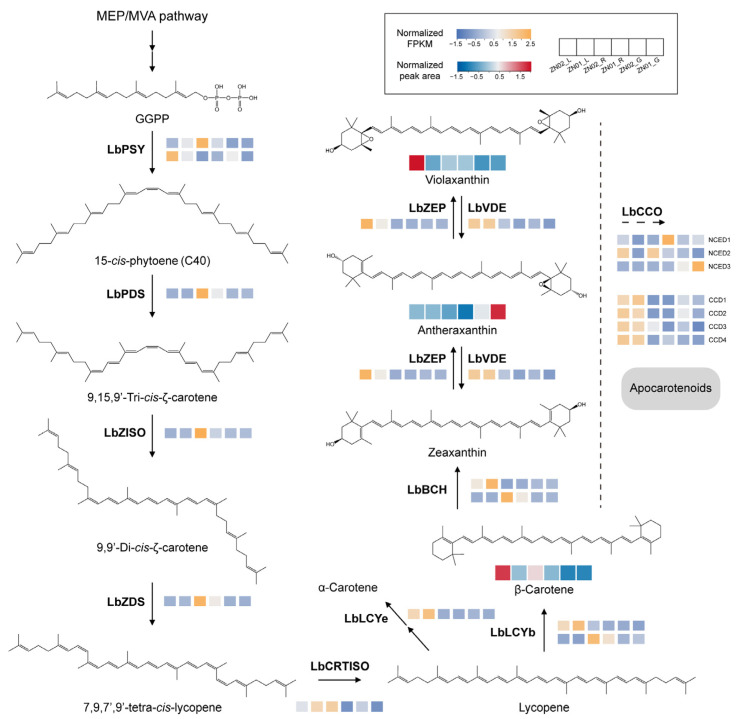
Biosynthesis pathway of carotenoids in *L*. *barbarum*. The color scale from purple to orange of the heatmaps below genes refers to the gene expression value from low to high. Heatmaps below the compounds indicate their relative quantification in different tissues, with content ranging from low (blue) to high (red). The double solid arrow represents multiple reaction steps. PSY, phytoene synthase; PDS, phytoene desaturases; ZISO, ζ-carotene isomerase; ZDS, ζ-carotene desaturases; CRTISO, carotenoid isomerase; LCYb, lycopene β-cyclase; LCYe, lycopene ε-cyclase; BCH, non-heme carotene hydroxylases; ZEP, zeaxanthin epoxidase; VDE, violaxanthin de-epoxidase; CCO, carotenoid cleavage oxygenase; CCD, carotenoid cleavage dioxygenase; NCED, 9-*cis*-epoxycarotenoid dioxygenase.

Previous research had indicated that *L. ruthenicum* fruits derive their color from anthocyanins, contrasting with *L. barbarum*, which is not abundant in anthocyanins (Table S2) [19,20]. Instead, carotenoids, particularly zeaxanthin dipalmitate, constitute the primary color substances in *L. barbarum*. The carotenoid biosynthesis pathway is evolutionarily conserved in plants [22]. Based on the metabolomic and transcriptomic profiles of mature fruits, immature fruits, and leaves from ZN02 and ZN01, we elucidated the carotenoid biosynthesis pathway in *L. barbarum* (Figure 3). The metabolomic analysis identified three C40 carotenoids in the pathway: β-carotene, atheraxanthin, and violaxanthin. β-carotene, crucial as a zeaxanthin precursor, plays a pivotal role in zeaxanthin dipalmitate synthesis. It was mostly found in the leaves and mature fruits of *L. barbarum*, with higher concentrations in ZN02. Atheraxanthin mainly accumulates in leaves and immature fruits, while violaxanthin generally accumulates in mature fruits. Beyond the known synthetic route, the metabolomic analysis detected 33 carotenoids, comprising 12 C40 carotenoids and 21 apocarotenoids (Table S2). Mature fruits exhibited the main accumulation of C40 carotenoids, whereas apocarotenoids were prevalent in immature fruits and leaves (Figure S5). The chlorophyll masking effect in leaves normally conceals carotenoids, resulting in color differences only between mature and immature fruits.

We also annotated 20 genes involved in the carotenoid biosynthesis pathway and examined their transcription levels using RNA-seq (Figure 3 and Table S10). Among these genes, *LbPSY1*, *LbPDS*, *LbZISO*, and *LbZDS* located upstream exhibited high expression levels in mature fruits. *LbCRTISO* showed high expression in leaves as well as in ZN02_R. We identified two key genes responsible for β-carotene synthesis: *LbLCYb1* and *LbLCYb2*. *LbLCYb1* was highly expressed in leaves, while *LbLCYb2* was expressed in mature fruits and hardly expressed in leaves. We also annotated a key gene *LbLCYe,* which is highly expressed in leaves, implying the accumulation of α-carotene in leaves. The precursor of zeaxanthin dipalmitate, derived from β-carotene catalyzed by BCH, *LbBCH1* is highly expressed in leaves and *LbBCH2* is highly expressed in mature fruits, mirroring the expression pattern of *LbLCYb*. *LbZEP* and *LbVDE* involved in the conversion of zeaxanthin, antheraxanthin, and violaxanthin were notably expressed in leaves. Chlorophyll synthesis genes were highly expressed in leaves, which masked the phenotype of carotenoids in leaves (Figure S6) [29]. Our analysis of transcriptional data from seven *LbCCOs* confirmed the distribution patterns of apocarotenoids. Regulation of β-carotenoid concentration is governed by carotenoid cleavage dioxygenase 4 (CCD4) [30], with significantly higher expression observed in the leaves compared to fruits. Furthermore, we also found that the expression of *CCD4*-related genes (*LbCCD1* and *LbCCD2*) was inhibited in mature fruits.

### 2.4. LbGT/GH May Be Involved in the Final Formation of AA-2βG

Initially, we indicated the potential significant role of glycosyltransferase/glycoside hydrolase (GT/GH) in the AA-2βG biosynthesis pathway in mature fruits. Most of the β-glucosidase (BGLU) and β-galactosidase (BGAL) in plants belong to GH1 and GH35, respectively (https://www.cazy.org, accessed on 22 December 2024). A critical aspect of this glycosylation process is the distinction in the α/β configuration of the linkage between AA and the sugar moiety. We hypothesized the potential involvement of UGT, GH1, and GH35 families in the last glycosylation step.

**Figure 4:**
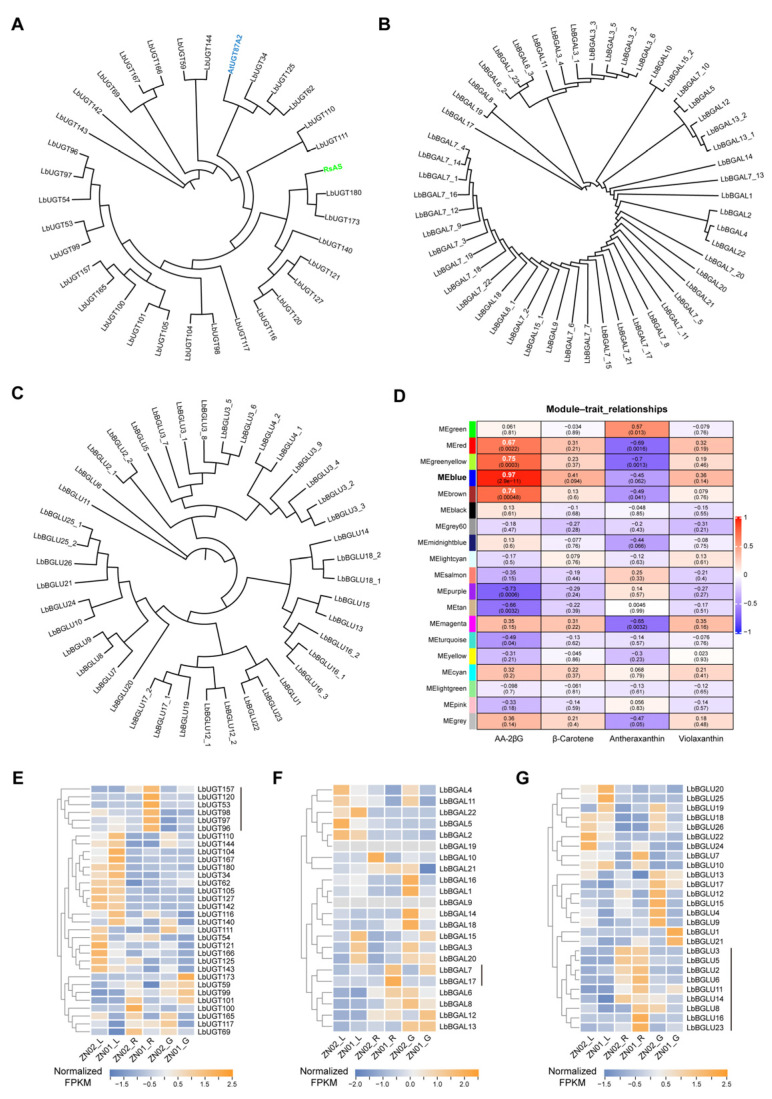
Screening of LbGT/GH candidates for AA-2βG biosynthesis final step. (**A**–**C**) Phylogenetic analysis of *L. barbarum* UGTs with AtUGT87A2 and RsAs homologs (**A**), BGALs (**B**), and BGLUs (**C**). (**D**) Module–trait relationships between 19 modules and AA-2βG, β-carotene, antheraxanthin, and violaxanthin. The Pearson correlation coefficient (PCC) of each module with different metabolites are given and colored according to the score, ranging from low (blue) to high (red). The numbers in parentheses represent the *p*-value. (**E**–**G**) Expression levels of candidate *LbUGTs* (**E**), *LbBGALs* (**F**), and *LbBGLUs* (**G**). The blue letters represent AtUGT87A2, while the green letters denote RsAs, as shown in Figure S7. UGT, UDP-glycosyltransferase; BGAL, β-galactosidase; BGLU, β-glucosidase.

Most UGTs primarily facilitate β-glycosylation, whereas only a few, such as the flavonoid α-L-rhamnosyltransferase, participate in α-glycosylation processes (https://www.cazy.org/GT1.html, accessed on 22 December 2024). We identified 181 UGTs from the genome of *L. barbarum* (PRJNA640228) and conducted a phylogenetic analysis comparing them with *A. thaliana* UGT (AtUGT) and RsAS (Figure S7). We screened 32 LbUGTs related to AtUGT87A2 and RsAS and assessed the gene expression levels of them by heatmap (Figure 4A,E). Although *LbUGT53*, *LbUGT96*, *LbUGT97*, *LbUGT98*, *LbUGT120*, and *LbUGT157* exhibited comparable expression patterns to AA-2βG, with higher expression in ZN01_R than in ZN02_R, only *LbUGT157* demonstrated a relatively high expression level (FPKM value > 20) (Table S11), potentially indicating its role in AA-2βG biosynthesis.

5,6-O-(isopropylidene) ascorbic acid has a similar configuration with AA near the 2-OH, which can synthesize 2-O-(β-D-Galactopyranosyl) ascorbic acid in the presence of β-galactosidase (Figure S8) [27]. Therefore, we identified the GH35 family in *L. barbarum*, screening 22 LbBGALs, including multiple transcripts of *LbBGAL3*, *LbBGAL6*, *LbBGAL7*, *LbBGAL13*, and *LbBGAL15* (Figure 4B). Notably, only *LbBGAL7* and *LbBGAL17* displayed similar expression patterns with AA-2βG, though at relatively lower levels (Figure 4F and Table S11).

Cellulase is a complex enzyme composed of β-glucosidase, endo-β-1,4-glucanase, and exo-β-1,4-glucanase. Therefore, we speculated that β-glucosidase mainly governs the glycosylation process of AA. Subsequently, our screening revealed 26 LbBGLUs, with *LbBGLU2*, *LbBGLU3*, *LbBGLU4*, *LbBGLU12*, *LbBGLU16*, *LbBGLU17*, *LbBGLU18*, and *LbBGLU25* exhibiting numerous transcripts (Figure 4C). Heatmap analysis showed that *LbBGLU2*, *LbBGLU3*, *LbBGLU5*, *LbBGLU6*, *LbBGLU14*, and *LbBGLU23* were highly expressed in mature fruits. Significantly, the expression levels of *LbBGLU2*, *LbBGLU6*, and *LbBGLU23* in ZN01_R surpassed those in ZN02_R, displaying positive correlations with AA-2βG content (Figure 4G and Table S11).

To advance our investigation into LbGT/GH within the AA-2βG biosynthesis pathway, we employed weighted gene co-expression network analysis (WGCNA) to analyze the interrelation between co-expressed genes and AA-2βG. Reserved genes were divided into 19 modules, which were indicated in different colors (Figure S9A). The Pearson correlation coefficient (PCC) revealed that AA-2βG was highly correlated with the genes of the MEblue module, with correlation coefficients greater than 0.95. The MEblue module contained three *LbBGLUs* (2, 6, and 23), nine *LbUGTs* (4, 58, 65, 67, 70, 84, 96, 113, and 135), and four AA pathway structure genes (*LbPMI3*, *LbGR2*, *LbAPX3*, and *LbAPX8*) (Figure 4D and Figure S9B; Table S12).

Based on the well-defined glycosylation transfer mechanism of UGT [31], we conducted structure predictions [32] and molecular docking [33] for 10 candidate LbUGTs. The result showed that LbUGT70 facilitated the physical proximity between UDP and AA (2.9 Å), with LbUGT58, LbUGT65, LbUGT67, LbUGT84, and LbUGT157 also demonstrating closer physical distances (3.1–4.1 Å) and lower affinity values (−5.2 to −6.0 kcal/mol) (Figure S10A–F). LbUGT4, which is complex with UDPG, possibly presents a more realistic scenario compared to other UDP complexes, featuring a physical distance of 4.6 Å and an affinity value of −4.6 kcal/mol (Figure S10G). However, LbUGT96, LbUGT113, and LbUGT135 are constrained by the configuration and position of phosphate groups of UDP, hindering the attack by the 2-OH of AA, resulting in relatively further physical distances (5.4–5.5 Å) (Figure S10H–J).

Other modules with high PCCs, such as MEgreenyellow, MEbrown, and MEred, contained seven *LbUGTs*, one *LbBGLU*, one *LbBGAL*, and six genes in the AA-2βG biosynthesis pathway (*LbGGP2*, *LbPGI1*, *LbGME1*, *LbMDHAR5*, *LbDHAR1*, and *LbGalLDH*) (Figure 4D and Table S12). These identified genes are present as potential candidates responsible for catalyzing AA-2βG biosynthesis. Furthermore, an analysis of the relation between co-expressed genes and β-carotene, antheraxanthin, and violaxanthin did not unearth highly correlated modules in the results. Interestingly, the six structural genes for carotenoid biosynthesis (*LbPSY1*, *LbPDS*, *LbZISO*, *LbZDS*, *LbLCYb2*, and *LbBCH2*) were primarily concentrated in the MEbrown module (Figure 4D and Table S12).

### 2.5. Validation of the Differentially Expressed Genes in AA-2βG and Carotenoid Biosynthesis Pathway by RT-qPCR

**Figure 5:**
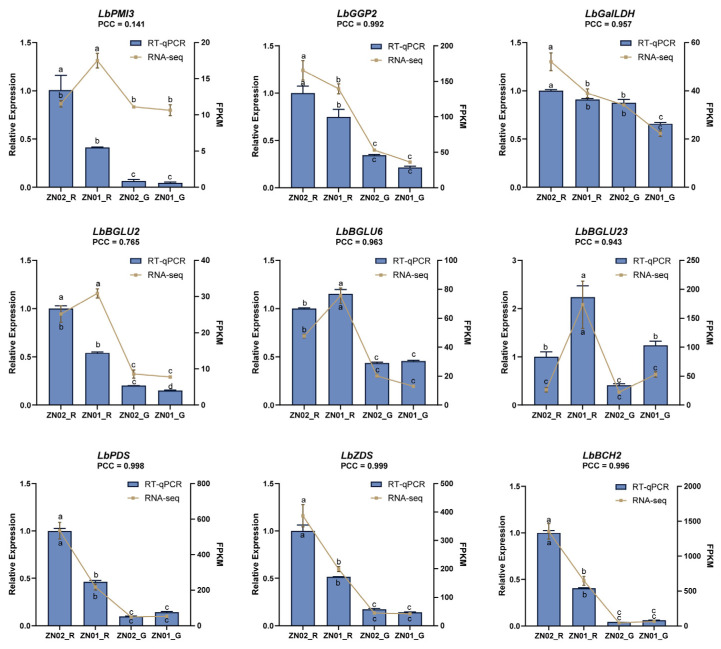
RT-qPCR results of *LbPMI3*, *LbGGP2*, *LbGalDH*, *LbBGLU2*, *LbBGLU6*, *LbBGLU23*, *LbPDS*, *LbZDS*, and *LbBCH2* in mature and immature fruits of two cultivars of *L*. *barbarum*, ZN01 and ZN02. The column chart displayed the RT-qPCR results for each gene, while the line chart illustrated the FPKM values by RNA-seq. The Pearson correlation coefficient (PCC) between FPKM value and RT-qPCR is located below the gene ID. The lowercase letters indicate significance levels at *p* ≤ 0.05, as determined by Tukey’s test.

In order to further characterize the functions of structural genes from the AA-2βG and carotenoid biosynthesis pathways, their expression levels in various tissues were analyzed by RT-qPCR (Figure 5). *LbEF1α* served as the reference gene [34] for verifying the cDNA of ZN02_R, ZN01_R, ZN02_G, and ZN01_G through RT-qPCR. The results revealed that, in the upstream AA-2βG synthesis, the FPKM values of *LbGGP2* and *LbGalLDH* aligned consistently with the RT-qPCR results (PCC > 0.95). Conversely, *LbPMI3* displayed higher expression in ZN02_R in the RT-qPCR results than indicated by the FPKM value (PCC = 0.141), mirroring the expression pattern of *LbGGP2* and *LbGalLDH*. Similarly, *LbBGLU2* exhibited a similar discrepancy (PCC = 0.765). Notably, the high expression of *LbBGLU6* and *LbBGLU23* showed the same trend in RNA-seq and RT-qPCR (PCC > 0.95), which further indicated the importance of the glycosylation process for AA-2βG biosynthesis. In the carotenoid biosynthesis pathway, the FPKM and RT-qPCR expression of *LbPDS*, *LbZDS*, and *LbBCH2* also had the same trend (PCC > 0.99), associated with the accumulation of β-carotene.

## 3. Discussion

AA accumulates highly in the fruits of some plants [35], with more stable AA-2βG reported in only a few species, such as *Malus sylvestris*, *Prunus armeniaca*, *Actinidia arguta* [36], and *L. barbarum* [11]. Quantitative variation of AA-2βG in goji berry cultivars exhibited a concentration range of 6.27–13.2 mg·g^−1^ dry weight [37]. It was estimated that AA-2βG accounts for 0.5% of dried *L. barbarum* fruits, equivalent to the AA content of fresh lemons [8]. We conducted AA-2βG content tests on *L. barbarum* fruits from seven different cultivars and found that ZN01_R had a higher AA-2βG content (30.42 mg·g^−1^). While *L*. *barbarum* leaves showed relatively low accumulation levels of AA-2βG, they demonstrated a distinct agronomic advantage through their extended harvesting window [38]. We did not detect AA-2βG in the leaves by UPLC, although it was detected in the metabolic data, accounting for only about 0.1% compared to mature fruits. Similarly, AA-2βG was not detected in immature fruits by UPLC. The varietal differences in AA-2βG distribution, along with high spatiotemporal specificity, prompted further investigation into more metabolic and gene transcription differences behind this variation.

The upstream of the AA-2βG biosynthesis pathway is the conserved AA biosynthesis pathway, with the Smirnoff–Wheeler pathway being the clearest. We constructed the Smirnoff–Wheeler homologous pathway in *L. barbarum*, but not all metabolites and genes in the pathway showed co-expression with varietal and spatiotemporal differences in AA-2βG content. The accumulation patterns of GDP-α-D-mannose and AA-2βG differ, potentially attributed to the significant expression of *LbGME1* in mature fruits, with FPKM values exceeding 400 (ZN01_R) and 900 (ZN02_R). This enhanced expression leads to the rapid conversion of GDP-α-D-mannose to GDP-β-L-galactose. Meanwhile, the diverse metabolic pathways of GDP-α-D-mannose (www.genome.jp/pathway/lbb00051, accessed on 17 December 2024) may also lead to complex changes in its content. Of all genes participating in AA biosynthesis, *LbNMNAT*, *LbPGI1*, *LbPMI3*, *LbGME1*, *LbGGP2*, and *LbGalLDH* were related to AA-2βG content in mature fruits, but WGCNA results indicated only *LbPMI3* met the AA-2βG accumulation criteria between ZN01_R and ZN02_R. Although our RT-qPCR results provided transcriptional-level explanations for differential AA-2βG accumulation between mature and immature fruits, paradoxically, *LbPMI3*, *LbGGP2*, and *LbGalLDH* expression patterns in ZN01_R and ZN02_R exhibited inverse correlations with the final AA-2βG concentrations. We also analyzed the supplementary pathways of reported AA biosynthesis and AA-recycling structural genes and found that *LbGalUR* (7, 8, and 9), *LbMIOX* (1 and 2), *LbDHAR1*, and *LbMDHAR* (1, 2, 3, 4, and 5) may be involved in AA-2βG biosynthesis. There were many AA-recycling structural genes expressed in the leaves, which also cannot explain the low AA-2βG content in leaves. The expression of these structural genes fully illustrates the complexity of monosaccharide metabolism and highlights the importance of the final-step glycosylation process in AA-2βG biosynthesis. Previous studies have showed that AA content in *L. barbarum* berries ranges from 2.39 to 48.94 mg·100 g^−1^ (fresh weight) [39]. Notably, a comparative analysis revealed significantly reduced AA accumulation during the red-ripening stage in *Lycium chinense* fruits [18]. While AA mediates conserved redox-related physiological functions across plant species [40], our study revealed that the mature fruits predominantly accumulate AA-2βG, with AA exhibiting low abundance or even being undetectable [8]. Glycosylation is integral to numerous biosynthetic and signaling networks that contribute to the maintenance of redox homeostasis. This process plays a direct role in regulating the homeostasis of reactive oxygen species (ROS) through the glycosylation of specific metabolites. Moreover, it modulates plant hormones by glycosylation, thereby influencing their activity [41]. The glycosylation process, which may be similar to the AA-recycling process, plays a crucial role in maintaining AA homeostasis in *L. barbarum*.

Carotenoids are another important nutritional component in *L. barbarum*. By comparing the content and type differences of carotenoids in immature fruits, mature fruits, and the leaves of different cultivars, we found that carotenoids are primarily present in the form of apocarotenoids in leaves and immature fruits, while in mature fruits they mainly existed in the form of C40 carotenoids. The concurrent high expression of *LbLCYb2* and *LbBCH2* in mature fruits, coupled with the downregulation of *CCD4*-related genes (*LbCCD1* and *LbCCD2*), provides insight into the distinctive distribution patterns of apocarotenoids among varied tissues and fruits at varying ripeness stages. Notably, the NCEDs mainly participate in ABA synthesis [42], with *LbNCED2* showing a high expression level in both the leaves and mature fruits of ZN02. Additionally, differential expression of pathway structural genes can directly explain the differences in β-carotene content between mature and immature fruits. The *L. barbarum* fruit contains high levels of carotenoids and undetectable anthocyanins, while the *L. ruthenicum* fruit has abundant anthocyanins and a very low amount of carotenoids [20]. The color change during the maturation process of *L. barbarum* was different from *L. ruthenicum* and followed a carotenoid accumulation pattern. Ultrastructural analysis indicated that the number of tubular chromoplasts determines the pattern of color accumulation [43]. This difference in accumulation pattern was accompanied by differences in AA-2βG content, leading us to speculate on possible correlations. Some evidence suggests a positive correlation between AA, the precursor of AA-2βG, and anthocyanin accumulation. In high-light acclimation, wild-type *A. thaliana* accumulated more anthocyanins than ascorbate-deficient mutants *vtc1*, *vtc2*, and *vtc3* [44]. At the transcriptional regulation level, *SlAN2* played a crucial role in anthocyanin biosynthesis, and its overexpression can activate the inositol pathway, upregulating *DHAR* and *APX*, and accumulating AA content [45]. In apples, MdMYB1, which participates in forming the MBW complex, directly activated *MdDHAR* expression, promoting AA accumulation [46]. AA is directly related to carotenoid biosynthesis. In the chloroplast, AA is speculated to be associated with photosynthesis and photoprotection, where thylakoid lumen AA acts as a co-factor for violaxanthin de-epoxidase (VDE) in the xanthophyll cycle [47,48]. Our results showed that the expression level of *LbVDE* in ZN02_R was higher than in ZN01_R, resulting in a high accumulation of antheraxanthin in ZN02_R, but the content of AA-2βG was higher in ZN01_R. Thus, the association of AA with anthocyanins and carotenoids in plants is complex, potentially involving multiple mechanisms between different tissues, and may also be related to the form of AA-2βG.

For the final step of AA-2βG biosynthesis, we referred to the case where hydroquinone can be glycosylated to produce α and β configurations and focus on UGT [22,23]. UGT primarily catalyzes the formation of β-glucosides, as demonstrated by a study showing that overexpressing of *UGT87A2* in *A. thaliana* increased AA-2βG content in plants [24]. Unfortunately, our screening results indicate that most genes homologous to *RsAs* and *AtUGT87A2* have inconsistent accumulation patterns with AA-2βG content, and only *LbUGT157* with a relatively high expression level (FPKM > 20) can be further validated. Notably, WGCNA analyzed the expression patterns of all one-hundred-and-eighty-one *LbUGTs*, and we identified nine new *LbUGTs* co-expressed with AA-2βG content and having relatively high expression levels, and the activities were predicted through molecular docking. The identified 10 *LbUGTs* are likely to have key functions in the biosynthesis of AA-2βG and the maintenance of AA homeostasis, aiming to enhance our comprehension of the specific distribution of AA-2βG in *L. barbarum*. All plant β-galactosidases (BGALs) are categorized within the GH35 family and are known to exhibit β-glucosylating activity [49]. Furthermore, through showcasing the transglycosylation activities and reverse hydrolysis of specific BGALs in vitro, it has been suggested that they might play a biosynthetic role in vivo [50,51]. Due to the structural resemblance of glucose to galactose, it is hypothesized that BGALs might possess analogous β-glycosylation functions towards AA [27]. Unfortunately, most of the BAGLs we screened have low expression, losing the potential for subsequent verification. Enzymes related to cellulase from *T. virus*, *A. niger*, and *T. reesei* have been demonstrated to effectively convert AA to AA-2βG [28]. An avenacin biosynthesis study showed the enzyme that adds the final 1,4-linked D-glucose is not a UGT, but rather a sugar transferase belonging to GH1 [52]. We speculate that β-glucosidase (BGLU) plays a primary role in this process. Combining screening results with WGCNA, we identified *LbBGLU2*, *LbBGLU6*, and *LbBGLU23* whose expression patterns and levels aligned with expectations and conducted RT-qPCR validation. This provides a foundation for completing the critical steps of AA-2βG biosynthesis.

## 4. Materials and Methods

### 4.1. Plant Materials

Seedlings of seven cultivars of *L. barbarum* have been collected in Zhongning County, Ningxia, China, and were named ZN01, ZN02, ZN03, ZN04, ZN05, ZN06, and ZN07. These seedlings were from cuttings and have been planted in our laboratory’s experimental field located in Daxing District, Beijing, China (39°37′54.120″ N, 116°20′15.191″ E). The temperature during the fruit growth period ranges from 23 to 32 °C. Fruits of three four-year cultivars (ZN04, ZN05, and ZN07) and four three-year cultivars (ZN01, ZN02, ZN03, and ZN06) were harvested in August 2024 and were stored at −80 °C until use. Leaves (L), immature fruits (green, G), and mature fruits (red, R) of two selected cultivars, ZN01 and ZN02, were later collected after the content determination of AA-2βG. According to Fatchurrahman’s classification method [53], immature fruits are categorized as stage 1, while mature fruits are classified as stage 5. Mature fruits are deemed suitable for eating and consumption at this stage. Samples of whole fruits and leaves were wrapped in tin foil paper, placed in liquid nitrogen, and stored at −80 °C until use. Each sample had three biological replicates and was divided into two parts for metabolome and transcriptome analyses.

### 4.2. Reagents

2-O-β-D-glucopyranosyl-L-ascorbic acid was purchased from Chengdu Push Bio-technology Co., Ltd., Chengdu, China. Ammonium acetate was purchased from Shanghai Aladdin Biochemical Technology Co., Ltd., Shanghai, China. Acetonitrile was purchased from Thermo Fisher Scientific, Waltham, MA, USA. Other reagents were analytical grade.

### 4.3. Determination of AA-2βG Content

Samples of whole fruits and leaves were vacuum-freeze-dried. Three fruits of each cultivar were taken and crushed, and water was added in a 1:25 (*w:v*) ratio. The mixture was sonicated for 25 min. Before loading the sample, the extract was diluted five times and filtered through a 0.22 μm microporous membrane.

A Shimadzu Nexera UPLC LC-20A system (Shimadzu, Kyoto, Japan) equipped with a binary pump, an auto-sampler, a diode-array detector (DAD), and a column temperature controller was used for quantitative analysis. Chromatographic separation of AA-2βG was carried out on an ACQUITY UPLC Glycan BEH Amide Column, 130 Å, 1.7 µm, 2.1 mm × 150 mm. The mobile phase is: 0.05 mol·L^−1^ ammonium acetate solution (A): acetonitrile (B); 0–10 min 78% B to 65% B; 10–12 min 65% B to 55% B; 12–15 min 55% B; 15–20 min 55% B to 78% B. The flow rate is 0.30 mL·min^−1^, the detection wavelength is 260 nm, and the column temperature is 25 °C.

### 4.4. Non-Targeted Metabolomics Analysis

Leaves (L), immature fruits (green, G), and mature fruits (red, R) of two selected cultivars, ZN01 and ZN02, were freeze-dried. Homogenization beads were added to 25 mg ± 1 mg of the sample, and 1000 μL of a pre-cooled extraction solvent (methanol: acetonitrile: water = 2:2:1, *v*/*v*/*v*) was added. The extraction solvent contained isotopically labeled internal standards. After 30 s of vortexing, the mixture was homogenized for 4 min at 35 Hz in a homogenizer before being moved to an ice-water bath and sonicated for 5 min. After performing this three times, it was left to stand at −40 °C for an hour. After adding 400 μL of the liquid to the wells of a protein precipitation plate, the plate and collecting plate were put in a positive pressure apparatus set to 6 psi for 120 s. The filtrate was then gathered and subjected to machine analysis. For all samples, take an equal amount of supernatant and mix to create a quality control (QC) sample for machine analysis.

For non-polar metabolites, the Vanquish (Thermo Fisher Scientific, Waltham, MA, USA) ultra-high-performance liquid chromatography (UPLC) system, with the Phenomenex Kinetex C18 (2.1 mm × 50 mm, 2.6 μm) liquid chromatography column, was used to chromatographically separate the target compounds. The mobile phase A for the liquid chromatography is an aqueous phase containing 0.01% acetic acid, and the mobile phase B is isopropanol: acetonitrile (1:1, *v*/*v*). Sample tray temperature was 4 °C, and injection volume was 2 μL. The Orbitrap Exploris 120 mass spectrometer is capable of performing both MS1 and MS2 data acquisition under the control of the software (Xcalibur, version 4.4, Thermo Fisher Scientific, Waltham, MA, USA). The detailed parameters were as follows: sheath gas flow rate: 50 Arb; aux gas flow rate: 15 Arb; capillary temperature: 320 °C; full MS resolution: 60,000; MS/MS resolution: 15,000; collision energy: SNCE 20/30/40; and spray voltage: 3.8 kV (positive) or −3.4 kV (negative).

For polar metabolites, LC-MS/MS analyses were performed using a UPLC system with a waters ACQUITY UPLC BEH amide (2.1 mm × 50 mm, 1.7 μm) coupled to an Orbitrap Exploris 120 mass spectrometer (Orbitrap MS, Thermo Fisher Scientific, Waltham, MA, USA). The mobile phase consisted of 25 mmol·L^−1^ ammonium acetate and 25 mmol·L^−1^ ammonia hydroxide in water (pH = 9.75) (A) and acetonitrile (B). The auto-sampler temperature was 4 °C, and the injection volume was 2 μL. The Orbitrap Exploris 120 mass spectrometer was used for its ability to acquire MS/MS spectra in information-dependent acquisition (IDA) mode in the control of the acquisition software (Xcalibur, Thermo Fisher Scientific, Waltham, MA, USA). In this mode, the acquisition software continuously evaluates the full scan MS spectrum. The ESI source conditions were set as follows: sheath gas flow rate as 50 Arb; aux gas flow rate as 15 Arb; capillary temperature as 320 °C; full MS resolution as 60,000; MS/MS resolution as 15,000; collision energy as SNCE 20/30/40; and spray voltage as 3.8 kV (positive) or −3.4 kV (negative), respectively.

In order to find differentially accumulated metabolites, supervised orthogonal projections to latent structures discriminate analysis (OPLS-DA) was applied. Furthermore, the value of variable importance in the projection (VIP) of the first principal component in OPLS-DA was obtained. It summarizes the contribution of each variable to the model. The metabolites with VIP > 1 and *p* < 0.05 (Student’s *t*-test) were considered as differentially accumulated metabolites. Metabolites were annotated and classified according to the Kyoto Encyclopedia of Genes and Genomes (KEGG) database. Network analysis was conducted using R (network v1.16.1 and igraph v1.2.6).

### 4.5. Transcriptome Analysis

Total RNA was extracted using the Trizol reagent (Thermo Fisher Scientific, Waltham, MA, USA, 15596018) following the manufacturer’s procedure. High-quality RNA samples with a RIN number > 7.0 were utilized to build the sequencing library, and the Bioanalyzer 2100 and RNA 6000 Nano LabChip Kit (Agilent, Palo Alto, CA, USA) were employed to analyze the total RNA amount and purity. Following the extraction of total RNA, a Dynabeads Oligo (dT) (Thermo Fisher Scientific, Waltham, MA, USA) was used to purify mRNA from total RNA (5 μg) in two rounds. After purification, the mRNA was broken up into small pieces using divalent cations at a high temperature (94 °C for 5–7 min) using the Magnesium RNA Fragmentation Module (NEB, Ipswich, MA, USA). Subsequently, SuperScript^TM^ II Reverse Transcriptase (Invitrogen, Carlsbad, CA, USA) reverse-transcribed the cleaved RNA fragments to produce the cDNA, which was then utilized to synthesize U-labeled second-stranded DNAs using *E. coli* DNA polymerase I (NEB, Ipswich, MA, USA), RNase H (NEB, Ipswich, MA, USA), and dUTP Solution (Thermo Fisher Scientific, Waltham, MA, USA). Each strand’s blunt ends were then treated with an A-base to prepare them for ligation to the indexed adapters. To ligate the adapter to the A-tailed fragmented DNA, each adapter included a T-base overhang. AMPure XP beads were used for size selection after dual-index adapters were ligated to the fragments. Following the U-labeled second-stranded DNAs’ treatment with the heat-labile UDG enzyme (NEB, Ipswich, MA, USA), the ligated products were amplified using PCR under the following conditions: initial denaturation at 95 °C for 3 min; 8 cycles of denaturation at 98 °C for 15 s, annealing at 60 °C for 15 s, and extension at 72 °C for 30 s; and then final extension at 72 °C for 5 min. The average insert size for the final cDNA library was 300 ± 50 bp. At last, we performed the 2 × 150 bp paired-end sequencing (PE150) on an Illumina Novaseq™ 6000 following the vendor’s recommended protocol.

Genes differential expression analysis was performed by DESeq2 software (v1.22.2) between two different groups (and by edgeR v3.22.5 between two samples). The genes with the parameter of false discovery rate (FDR) below 0.05 and absolute fold change ≥ 2 were considered differentially expressed genes (DEGs). DEGs were then subjected to enrichment analysis of KEGG pathways.

### 4.6. Gene Family Identification

The reported *A.thaliana* UGT, GH1, and GH35 family protein sequences were used as a query to blast the protein sequences in *L. barbarum*. Moreover, the hidden markov model (HMM) file (UGT: PF00201; GH1: PF00232; GH35: PF01301) of GT/GH was downloaded from Pfam (http://pfam.xfam.org/, accessed on 21 December 2024) and used as a query in HMM searches for candidate protein sequences in *L. barbarum*. To confirm the domain organization, the candidate protein sequences were characterized using the CD-Search Tool (https://www.ncbi.nlm.nih.gov/Structure/bwrpsb/bwrpsb.cgi, accessed on 21 December 2024), and the sequences lacking a conserved domain were eliminated manually. Phylogenetic analysis was constructed using the IQ-tree (http://www.iqtree.org/, accessed on 21 December 2024) program based on the muscle and the maximum likelihood (ML) method (best-fit model: UGT: LG + F + R7; GH1: LG + G4; GH35: WAG + I + G4) [54].

### 4.7. Weighted Gene Coexpression Network Analysis (WGCNA)

WGCNA was conducted using the WGCNA-shinyApp (https://github.com/ShawnWx2019/WGCNA-shinyApp, accessed on 26 December 2024). Co-expression modules were generated under default settings, with modifications only for the inclusion of 8000 reserved genes, a power selection of 14, a module cut height of 0.25, and a minimum module size of 30. Subsequently, genes showing similar expression profiles were grouped into the same modules. Finally, we established module-trait relationships by linking co-expressed gene modules with AA-2βG, β-carotene, antheraxanthin, and violaxanthin. TBtools-II (v2.138) was utilized for visualizing the FPKM matrix and metabolite relative abundance data in the form of a heatmap [55].

### 4.8. AlphaFold3 Structure Prediction and Molecular Docking

The structure model of LbUGTs were predicted by AlphaFold3 [32]. Molecular docking studies of LbUGTs with AA were accomplished by AutoDock Vina [33]. Ligands were pre-processed with proper charges and hydrogen atoms using AutoDock Tools 1.5.7. UDP or UDPG was packed into the predicted LbUGTs structure via superimposition. The reference PDB ID for UDP positions in LbUGT4, LbUGT58, LbUGT65, LbUGT67, LbUGT70, LbUGT84, LbUGT96, LbUGT113, LbUGT135, and LbUGT157 complex are 8i90, 5u6s, 7erx, 7erx, 7erx, 8hjo, 8wp5, 7zer, 7vej, and 8w53. The optimum docking complex with the best binding affinity was chosen to calculate the physical distances using PYMOL software (http://www.pymol.org, accessed on 30 January 2025).

### 4.9. Real-Time Quantitative PCR (RT-qPCR) Analysis

Total RNA extracted for transcriptome analysis was used for RT-qPCR analysis. cDNA was synthesized using a TransScript^®^ One-Step gDNA Removal and cDNA Synthesis SuperMix (TransGen Biotech, Beijing, China). Primer 6.0 was used to design primers. *LbEF1α* was selected as the housekeeping gene. RT-qPCR was performed on a Rotor-Gene Q (Qiagen, Hilden, Germany), with an annealing temperature set at 55 °C. The results of the RT-qPCR were analyzed using the 2^−ΔΔCt^ method. The expression level of each detected gene was normalized by using the housekeeping gene of *L. barbarum*. Selected gene and primer sequences used for RT-qPCR are listed in Table S13.

### 4.10. Statistical Analysis

Statistical analysis was performed using GraphPad Prism 9.0, and one-way ANOVA followed by Tukey’s post hoc test was utilized for statistical comparisons. Data are represented as means ± standard deviation (n ≥ 3).

## 5. Conclusions

In this study, we identified a *L. barbarum* cultivar with a high AA-2βG content and conducted a comparative transcriptional and metabolic analysis against the leaves, immature fruits, and mature fruits of normal cultivars. KEGG pathway analysis revealed enrichment in fructose and mannose metabolism (lbb00051) and carotenoid biosynthesis (lbb00906). We investigated the AA biosynthesis pathway and carotenoid metabolism in *L. barbarum*, discovering that the transcriptional and metabolic characteristics of the AA pathway were consistent with tissue-specific distribution. However, we observed discrepancies in the metabolic patterns among the mature fruits of different cultivars, prompting further investigation into the glycosylation process of AA. We identified 10 UGTs and 3 BGLUs that may be involved in the essential conversion of AA to AA-2βG. The activities of the UGTs were predicted through molecular docking, whereas the expression of BGLUs was validated by RT-qPCR. Additionally, AA is directly related to carotenoid biosynthesis, and transcription factors can simultaneously regulate the accumulation of AA and anthocyanins. The high enrichment of AA-2βG in goji berries, coupled with the low content of AA, led us to investigate the role of the glycosylation process. These findings provide the groundwork for further exploration of AA-2βG’s physiological functions and the relationship between nutrients in *L. barbarum*.

## Data Availability

The transcriptome sequencing raw data were deposited in the National Genomics Data Center (https://ngdc.cncb.ac.cn/) under the BioProject ID: PRJCA034309.

## Supplementary Materials

The following supporting information can be downloaded at: https://www.mdpi.com/article/10.3390/ijms26041558/s1.

## References

[1] Zhang Q., Chen W., Zhao J., Xi W. (2016). Functional Constituents and Antioxidant Activities of Eight Chinese Native Goji Genotypes. Food Chem..

[2] Kosińska-Cagnazzo A., Weber B., Chablais R., Vouillamoz J.F., Molnár B., Crovadore J., Lefort F., Andlauer W. (2017). Bioactive Compound Profile and Antioxidant Activity of Fruits from Six Goji Cultivars Cultivated in Switzerland. J. Berry Res..

[3] Gao Y., Wei Y., Wang Y., Gao F., Chen Z. (2017). *Lycium barbarum*: A Traditional Chinese Herb and a Promising Anti-Aging Agent. Aging Dis..

[4] Tyapkina D.Y., Kochieva E.Z., Slugina M.A. (2019). Vitamin C in Fleshy Fruits: Biosynthesis, Recycling, Genes, and Enzymes. Vavilov J. Genet. Breed..

[5] Smirnoff N., Wheeler G.L. (2024). The Ascorbate Biosynthesis Pathway in Plants Is Known, but There Is a Way to Go with Understanding Control and Functions. J. Exp. Bot..

[6] Naidu K.A. (2003). Vitamin C in Human Health and Disease Is Still a Mystery? An Overview. Nutr. J..

[7] Tao X., Su L., Wu J. (2019). Current Studies on the Enzymatic Preparation 2-O-α-D-Glucopyranosyl-L-Ascorbic Acid with Cyclodextrin Glycosyltransferase. Crit. Rev. Biotechnol..

[8] Toyoda-Ono Y., Maeda M., Nakao M., Yoshimura M., Sugiura-Tomimori N., Fukami H. (2004). 2-O-(β-D-Glucopyranosyl) Ascorbic Acid, a Novel Ascorbic Acid Analogue Isolated from *Lycium* Fruit. J. Agric. Food Chem..

[9] Kosińska-Cagnazzo A., Bocquel D., Marmillod I., Andlauer W. (2017). Stability of Goji Bioactives during Extrusion Cooking Process. Food Chem..

[10] Zhang Z., Liu X., Zhang X., Liu J., Hao Y., Yang X., Wang Y. (2011). Comparative Evaluation of the Antioxidant Effects of the Natural Vitamin C Analog 2-O-β-D-Glucopyranosyl-L-Ascorbic Acid Isolated from Goji Berry Fruit. Arch. Pharm. Res..

[11] Wang S.-F., Liu X., Ding M.-Y., Ma S., Zhao J., Wang Y., Li S. (2019). 2-O-β-d-Glucopyranosyl-L-Ascorbic Acid, a Novel Vitamin C Derivative from *Lycium barbarum*, Prevents Oxidative Stress. Redox Biol..

[12] Huang K., Dong W., Liu W., Yan Y., Wan P., Peng Y., Xu Y., Zeng X., Cao Y. (2019). 2-O-β-D-Glucopyranosyl-L-Ascorbic Acid, an Ascorbic Acid Derivative Isolated from the Fruits of *Lycium barbarum* L., Modulates Gut Microbiota and Palliates Colitis in Dextran Sodium Sulfate-Induced Colitis in Mice. J. Agric. Food Chem..

[13] Wheeler G.L., Jones M.A., Smirnoff N. (1998). The Biosynthetic Pathway of Vitamin C in Higher Plants. Nature.

[14] Agius F., González-Lamothe R., Caballero J.L., Muñoz-Blanco J., Botella M.A., Valpuesta V. (2003). Engineering Increased Vitamin C Levels in Plants by Overexpression of a D-Galacturonic Acid Reductase. Nat. Biotechnol..

[15] Wolucka B.A., Van Montagu M. (2003). GDP-Mannose 3′,5′-Epimerase Forms GDP-L-Gulose, a Putative Intermediate for the de Novo Biosynthesis of Vitamin C in Plants. J. Biol. Chem..

[16] Lorence A., Chevone B.I., Mendes P., Nessler C.L. (2004). *Myo*-Inositol Oxygenase Offers a Possible Entry Point into Plant Ascorbate Biosynthesis. Plant Physiol..

[17] Vargas J.A., Sculaccio S.A., Pinto A.P.A., Pereira H.D., Mendes L.F.S., Flores J.F., Cobos M., Castro J.C., Garratt R.C., Leonardo D.A. (2024). Structural Insights into the Smirnoff–Wheeler Pathway for Vitamin C Production in the Amazon Fruit Camu-Camu. J. Exp. Bot..

[18] Yin C., Xie H., Geng G., Li Z., Ma J., Wu X., Qiu Q.-S., Qiao F. (2024). Identification of Key Enzymes and Genes Modulating L-Ascorbic Acid Metabolism during Fruit Development of *Lycium chinense* by Integrating Metabolome, Transcriptome, and Physiological Analysis. Int. J. Mol. Sci..

[19] Ilić T., Dodevska M., Marčetić M., Božić D., Kodranov I., Vidović B. (2020). Chemical Characterization, Antioxidant and Antimicrobial Properties of Goji Berries Cultivated in Serbia. Foods.

[20] Liu Y., Zeng S., Sun W., Wu M., Hu W., Shen X., Wang Y. (2014). Comparative Analysis of Carotenoid Accumulation in Two Goji (*Lycium barbarum* L. and *L. ruthenicum* Murr.) Fruits. BMC Plant Biol..

[21] Hempel J., Schädle C.N., Sprenger J., Heller A., Carle R., Schweiggert R.M. (2017). Ultrastructural Deposition Forms and Bioaccessibility of Carotenoids and Carotenoid Esters from Goji Berries (*Lycium barbarum* L.). Food Chem..

[22] Sun T., Yuan H., Cao H., Yazdani M., Tadmor Y., Li L. (2018). Carotenoid Metabolism in Plants: The Role of Plastids. Mol. Plant.

[23] Liang M.-H., Zhu J., Jiang J.-G. (2018). Carotenoids Biosynthesis and Cleavage Related Genes from Bacteria to Plants. Crit. Rev. Food Sci. Nutr..

[24] Seo D.-H., Jung J.-H., Ha S.-J., Cho H.-K., Jung D.-H., Kim T.-J., Baek N.-I., Yoo S.-H., Park C.-S. (2012). High-Yield Enzymatic Bioconversion of Hydroquinone to α-Arbutin, a Powerful Skin Lightening Agent, by Amylosucrase. Appl. Microbiol. Biotechnol..

[25] Hefner T. (2002). Arbutin Synthase, a Novel Member of the NRD1β Glycosyltransferase Family, Is a Unique Multifunctional Enzyme Converting Various Natural Products and Xenobiotics. Bioorg. Med. Chem..

[26] von Saint Paul V. (2010). Stress Inducible Glycosyltransferases in *Arabidopsis thaliana* and Their Impact on Plant Metabolism and Defense Mechanisms. Ph.D. Thesis.

[27] Shimono Y., Hattori N., Donpou M. (1994). 2-o-beta-d-galactopyranosyl-l-ascorbic Acid or Its Salt, Its Production and Use Thereof. https://www.j-platpat.inpit.go.jp/c1801/PU/JP-H06-263790/11/en.

[28] Toyada-ono Y., Maeda M., Nakao M., Yoshimura M., Sugiura-tomimori N., Fukami H., Nishioka H., Miyashita Y., Kojo S. (2005). A Novel Vitamin C Analog, 2-O-(β-D-Glucopyranosyl) Ascorbic Acid: Examination of Enzymatic Synthesis and Biological Activity. J. Biosci. Bioeng..

[29] Frangedakis E., Yelina N.E., Billakurthi K., Hua L., Schreier T., Dickinson P.J., Tomaselli M., Haseloff J., Hibberd J.M. (2024). *MYB*-Related Transcription Factors Control Chloroplast Biogenesis. Cell.

[30] Li Y., Zhao H., Xia H.-X., Huang J., Ma N., Guo P., Liu Y.-P., Liu H.-L., Wang Y.-H., Lin N. (2024). Multiomics Analyses Provide Insights into the Genomic Basis of Differentiation among Four Sweet Osmanthus Groups. Plant Physiol..

[31] Kurze E., Wüst M., Liao J., McGraphery K., Hoffmann T., Song C., Schwab W. (2022). Structure–Function Relationship of Terpenoid Glycosyltransferases from Plants. Nat. Prod. Rep..

[32] Abramson J., Adler J., Dunger J., Evans R., Green T., Pritzel A., Ronneberger O., Willmore L., Ballard A.J., Bambrick J. (2024). Accurate Structure Prediction of Biomolecular Interactions with AlphaFold 3. Nature.

[33] Trott O., Olson A.J. (2010). AutoDock Vina: Improving the Speed and Accuracy of Docking with a New Scoring Function, Efficient Optimization, and Multithreading. J. Comput. Chem..

[34] Zhao J., Xu Y., Li H., An W., Yin Y., Wang B., Wang L., Wang B., Duan L., Ren X. (2024). Metabolite-based Genome-wide Association Studies Enable the Dissection of the Genetic Bases of Flavonoids, Betaine and Spermidine in Wolfberry (*Lycium*). Plant Biotechnol. J..

[35] Fenech M., Amaya I., Valpuesta V., Botella M.A. (2019). Vitamin C Content in Fruits: Biosynthesis and Regulation. Front. Plant Sci..

[36] Richardson A.T., McGhie T.K., Cordiner S.B., Stephens T.T.H., Larsen D.S., Laing W.A., Perry N.B. (2021). 2-O-β-D-Glucopyranosyl-L-Ascorbic Acid, a Stable Form of Vitamin C, Is Widespread in Crop Plants. J. Agric. Food Chem..

[37] Zhu B., Zhang W., Qin Y., Zhao J., Li S. (2022). Quality Evaluation of *Lycium barbarum* L. Fruits from Different Regions in China Based on 2-O-β-D-Glucopyranosyl-L-Ascorbic Acid. J. Food Compos. Anal..

[38] Bubloz C., Udrisard I., Micaux F., Piantini U., Amini-Rentsch L., Marti R., Andlauer W. (2020). The Vitamin C Analogue 2-O-β-D-Glucopyranosyl-L-Ascorbic Acid in Rhizomes, Stems and Leaves of *Lycium barbarum*: FH-HES Universities of Applied Sciences. Chimia.

[39] Teixeira F., Silva A.M., Delerue-Matos C., Rodrigues F. (2023). *Lycium barbarum* Berries (Solanaceae) as Source of Bioactive Compounds for Healthy Purposes: A Review. Int. J. Mol. Sci..

[40] Noctor G., Reichheld J.-P., Foyer C.H. (2018). ROS-Related Redox Regulation and Signaling in Plants. Semin. Cell Dev. Biol..

[41] Behr M., Neutelings G., El Jaziri M., Baucher M. (2020). You Want It Sweeter: How Glycosylation Affects Plant Response to Oxidative Stress. Front. Plant Sci..

[42] Tan B., Joseph L.M., Deng W., Liu L., Li Q., Cline K., McCarty D.R. (2003). Molecular Characterization of the *Arabidopsis* 9-*cis* Epoxycarotenoid Dioxygenase Gene Family. Plant J..

[43] Zeng S., Huang S., Yang T., Ai P., Li L., Wang Y. (2020). Comparative Proteomic and Ultrastructural Analysis Shed Light on Fruit Pigmentation Distinct in Two Lycium Species. Ind. Crops Prod..

[44] Page M., Sultana N., Paszkiewicz K., Florance H., Smirnoff N. (2012). The Influence of Ascorbate on Anthocyanin Accumulation during High Light Acclimation in *Arabidopsis thaliana*: Further Evidence for Redox Control of Anthocyanin Synthesis. Plant Cell Environ..

[45] Ye M., Wang D., Li R., Zhuang K., Wang H., Cao X., Qin T., Zhang H., Guo S., Wu B. (2025). *SlAN2* Overexpression Improves Cold Resistance in Tomato (*Solanum lycopersicum* L.) by Regulating Glycolysis and Ascorbic Acid Metabolism. Genomics.

[46] An J., Wang X., You C., Hao Y. (2020). The Anthocyanin Biosynthetic Regulator *MdMYB1* Positively Regulates Ascorbic Acid Biosynthesis in Apple. Front. Agric. Sci. Eng..

[47] Smirnoff N. (2000). Ascorbate Biosynthesis and Function in Photoprotection. Philos. Trans. R. Soc. Lond. B Biol. Sci..

[48] Müller-Moulé P., Havaux M., Niyogi K.K. (2003). Zeaxanthin Deficiency Enhances the High Light Sensitivity of an Ascorbate-Deficient Mutant of Arabidopsis. Plant Physiol..

[49] Chandrasekar B., Van Der Hoorn R.A.L. (2016). Beta Galactosidases in Arabidopsis and Tomato—A Mini Review. Biochem. Soc. Trans..

[50] Yoon J.-H., Ajisaka K. (1996). The Synthesis of Galactopyranosyl Derivatives with β-Galactosidases of Different Origins. Carbohydr. Res..

[51] Ahn Y.O., Zheng M., Bevan D.R., Esen A., Shiu S.-H., Benson J., Peng H.-P., Miller J.T., Cheng C.-L., Poulton J.E. (2007). Functional Genomic Analysis of *Arabidopsis thaliana* Glycoside Hydrolase Family 35. Phytochemistry.

[52] Orme A., Louveau T., Stephenson M.J., Appelhagen I., Melton R., Cheema J., Li Y., Zhao Q., Zhang L., Fan D. (2019). A Noncanonical Vacuolar Sugar Transferase Required for Biosynthesis of Antimicrobial Defense Compounds in Oat. Proc. Natl. Acad. Sci. USA.

[53] Fatchurrahman D., Amodio M.L., Valeria De Chiara M.L., Mastrandrea L., Colelli G. (2022). Characterization and Postharvest Behavior of Goji Berry (*Lycium barbarum* L.) during Ripening. Postharvest Biol. Technol..

[54] Minh B.Q., Schmidt H.A., Chernomor O., Schrempf D., Woodhams M.D., Von Haeseler A., Lanfear R. (2020). IQ-TREE 2: New Models and Efficient Methods for Phylogenetic Inference in the Genomic Era. Mol. Biol. Evol..

[55] Chen C., Wu Y., Li J., Wang X., Zeng Z., Xu J., Liu Y., Feng J., Chen H., He Y. (2023). TBtools-II: A “One for All, All for One” Bioinformatics Platform for Biological Big-Data Mining. Mol. Plant.

