# Supplementary material for: Integrated Multi-Omics Analysis Reveals Glycosylation Involving 2-O-β-D-Glucopyranosyl-L-Ascorbic Acid Biosynthesis in Lycium barbarum
Source: Int J Mol Sci. 2025 Feb 12;26(4):1558. doi: 10.3390/ijms26041558 (PMC11855784; doi:10.3390/ijms26041558)
Supplement: Supplementary file 1 [file ijms-26-01558-s001.zip › Supplementary Materials/Supplementary Figures S1-S10/Fig S10.pdf]

A

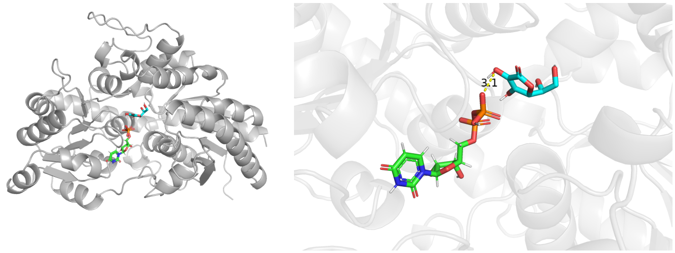

LbUGT58+UDP+AA (3.1 Å, −5.9 kcal/mol)

B

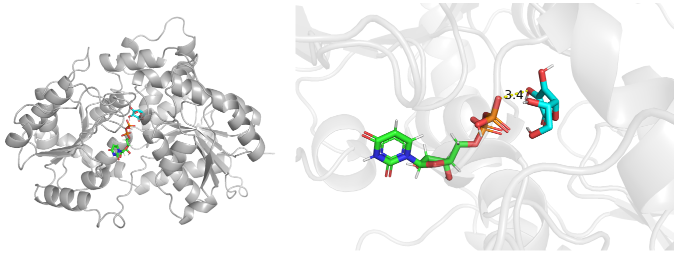

LbUGT65+UDP+AA (3.4 Å, −5.8 kcal/mol)

C

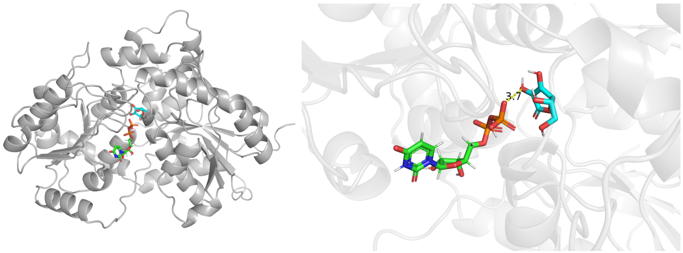

LbUGT67+UDP+AA (3.7 Å, −5.5 kcal/mol)

D

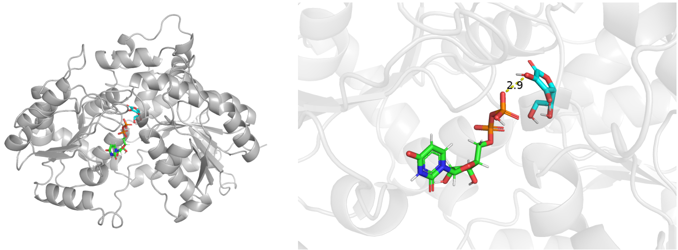

LbUGT70+UDP+AA (2.9 Å, −5.2 kcal/mol)

E

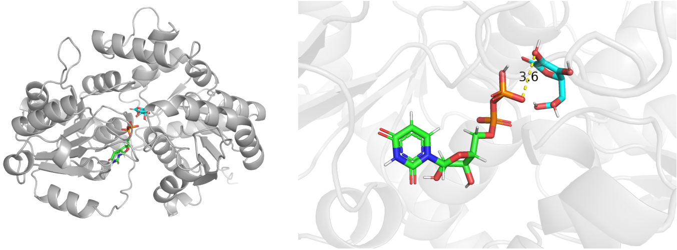

LbUGT84+UDP+AA (3.6 Å, −5.8 kcal/mol)

F

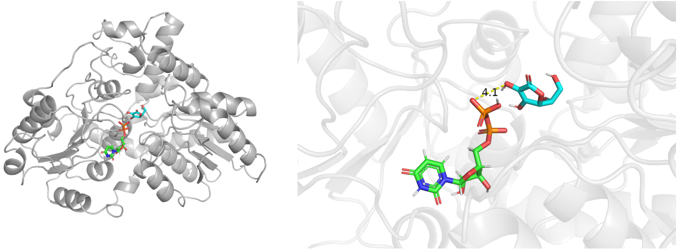

LbUGT157+UDP+AA (4.1 Å, −6.0 kcal/mol)

G

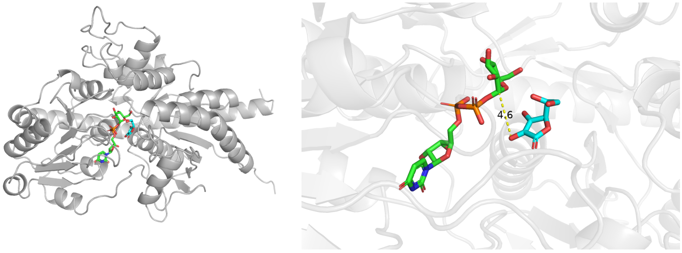

LbUGT4+UDPG+AA (4.6 Å, −4.6 kcal/mol)

H

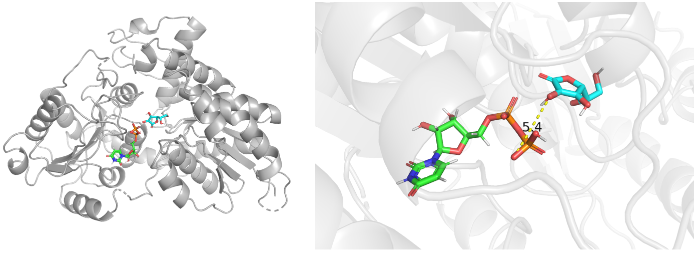

LbUGT96+UDP+AA (5.4 Å, −5.8 kcal/mol)

I

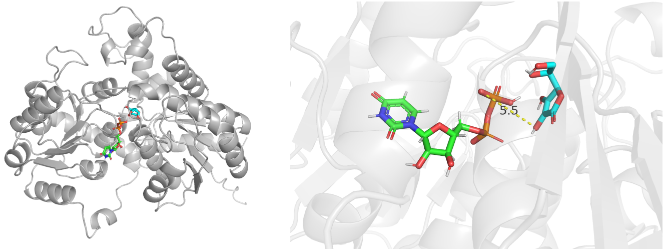

LbUGT113+UDP+AA (5.5 Å, −6.1 kcal/mol)

J

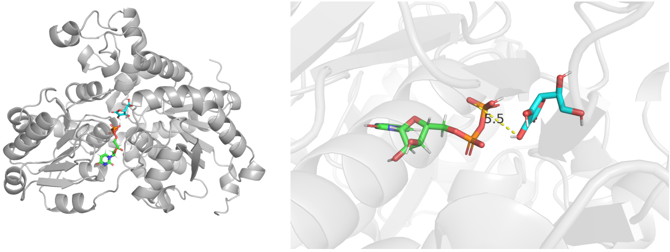

LbUGT135+UDP+AA (5.5 Å, −5.4 kcal/mol)
