# Supplementary material for: Integrated Multi-Omics Analysis Reveals Glycosylation Involving 2-O-β-D-Glucopyranosyl-L-Ascorbic Acid Biosynthesis in Lycium barbarum
Source: Int J Mol Sci. 2025 Feb 12;26(4):1558. doi: 10.3390/ijms26041558 (PMC11855784; doi:10.3390/ijms26041558)
Supplement: Supplementary file 1 [file ijms-26-01558-s001.zip › Supplementary Materials/Supplementary Figures S1-S10/Fig S5.pdf]

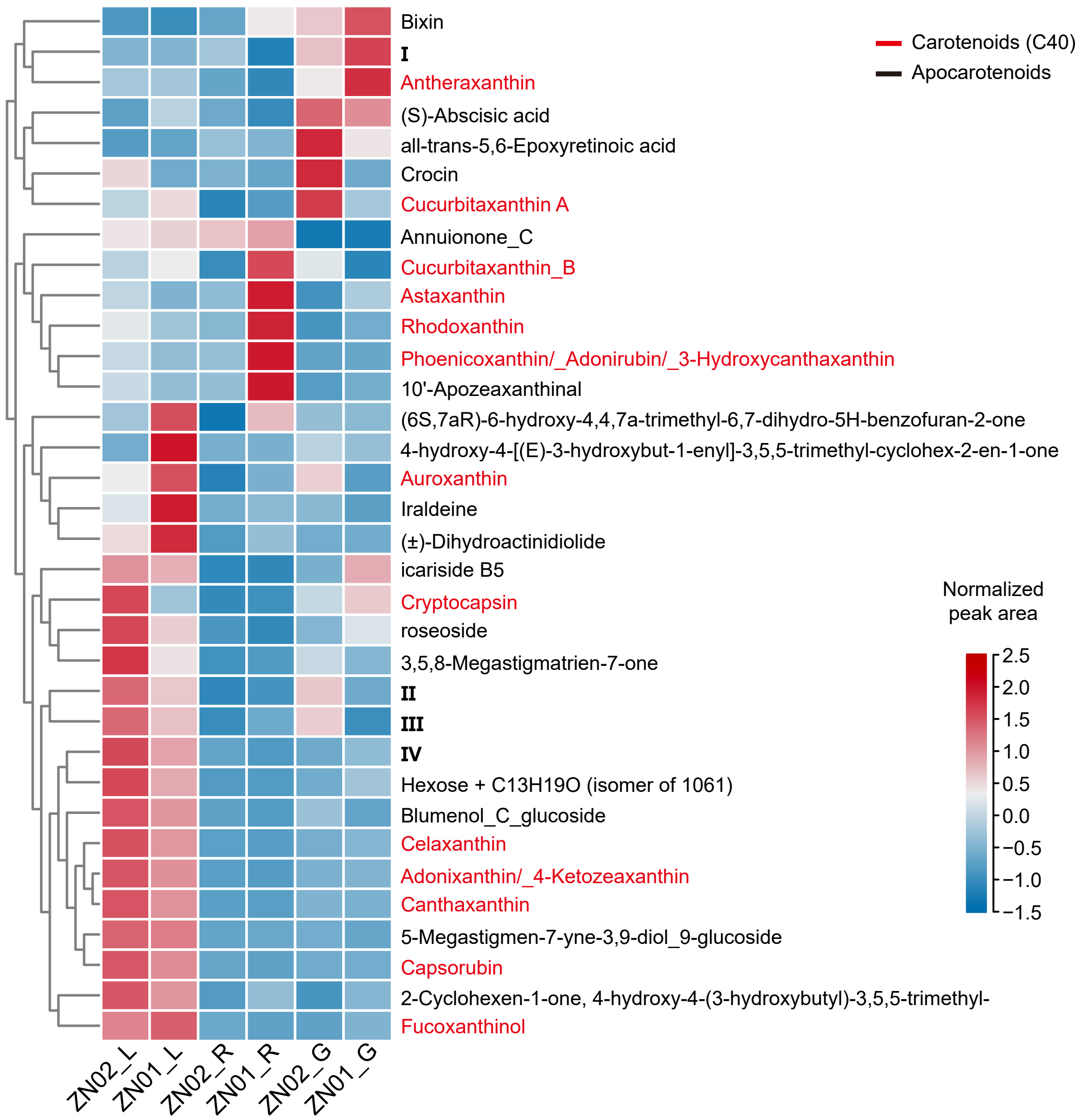

I: (2E,4E)-5-[8-hydroxy-1,5-dimethyl-3-[3,4,5-trihydroxy-6-(hydroxymethyl)tetrahydropyran-2-yl]oxy-6-oxabicyclo[3.2.1]octan-8-yl]-3-methyl-penta-2,4-dienoic acid

II: (2R,3R,4S,5S,6R)-2-[(E,1R)-3-[(1S,4S,6R)-1,4-dihydroxy-2,2,6-trimethyl-cyclohexyl]-1-methyl-allyloxy]-6-(hydroxymethyl)tetrahydropyran-3,4,5-triol

III: [3,4,5-trihydroxy-6-[1-methyl-3-(2,6,6-trimethyl-4-oxo-cyclohex-2-en-1-yl)propoxy]tetrahydropyran-2-yl]methyl 3,4,5-trihydroxybenzoate

IV: 4-[4-hydroxy-2,2,6-trimethyl-6-[(2S,3R,4S,5S,6R)-3,4,5-trihydroxy-6-(hydroxymethyl)tetrahydropyran-2-yl]oxy-cyclohexylidene]but-3-en-2-one
