# Supplementary material for: Integrated Multi-Omics Analysis Reveals Glycosylation Involving 2-O-β-D-Glucopyranosyl-L-Ascorbic Acid Biosynthesis in Lycium barbarum
Source: Int J Mol Sci. 2025 Feb 12;26(4):1558. doi: 10.3390/ijms26041558 (PMC11855784; doi:10.3390/ijms26041558)
Supplement: Supplementary file 1 [file ijms-26-01558-s001.zip › Supplementary Materials/Supplementary Figures S1-S10/Supplementary Figures' Captions.docx]

**Figure S1.** Principal component analysis results. (**A**) Transcriptome principal component analysis results. (**B**) Metabolome principal component analysis results.

**Figure S2.** Heatmap of genes involved in the other AA biosynthesis supplementary pathways and recycling pathways.

**Figure S3.** Network analysis of differentially expressed metabolites in ZN01 vs ZN02. The red line represents direct association.

**Figure S4.** KEGG enrichment analysis of (**A**) ZN01_R vs ZN01_L, (**B**) ZN01_R vs ZN01_G, (**C**) ZN02_R vs ZN02_L, and (**D**) ZN02_R vs ZN02_G. The name of pathways is indicated on the vertical axis, and the rich factor is indicated on the horizontal axis. The size of bubble represents the gene number, and the color represents the Q-value.

**Figure S5.** Heatmap of carotenoids identified from metabolome of leaves, mature (red), and immature (green) fruits of two cultivars of *L. barbarum*, ZN01 and ZN02.

**Figure S6.** Heatmap of gene expression level of genes involved in the metabolic pathway of Chlorophyll in leaves, mature (red), and immature (green) fruits of two cultivars of *L. barbarum*, ZN01 and ZN02.

**Figure S7.** The phylogenetic tree based on AtUGTs, RsAs, and LbUGTs. The blue branches represent the genes that cluster with AtUGT87A2, and the green branches represent the genes that cluster with RsAs. The red label indicates participation in the co-expression module.

**Figure S8.** Schematic diagram of the reaction where galactosidase catalyzes 5,6-O-(isopropylidene) ascorbic acid to 2-O-(β-D-Galactopyranosyl) ascorbic acid.

**Figure S9.** WGCNA for screening co-expression genes. (A) Top half of the image indicates the cluster tree, whereas the bottom half indicates the gene module. The genes that clustered together were grouped into the same module. (B) Heatmap of gene expression level of LbUGTs belonged to MEblue, MEgreenyellow, MEbrown, and MEred module.

**Figure S10.** Molecular docking analysis of candidate LbUGTs. The overall structure of LbUGTs in cartoon is displayed on the left side. UDP/UDPG and AA are present as green and blue sticks, respectively. The yellow dashed line represents the physical distance between UDP/UDPG and the 2-OH of AA. The text in parentheses below the figure represents the value of physical distance and the affinity of docking, respectively.
