# Supplementary figures and images for: Integrated Multi-Omics Analysis Reveals Glycosylation Involving 2-O-β-D-Glucopyranosyl-L-Ascorbic Acid Biosynthesis in Lycium barbarum
Source: Int J Mol Sci. 2025 Feb 12;26(4):1558. doi: 10.3390/ijms26041558 (PMC11855784; doi:10.3390/ijms26041558)

**A**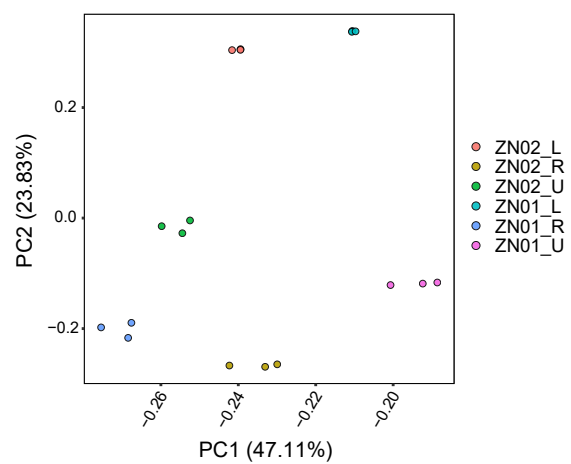**B**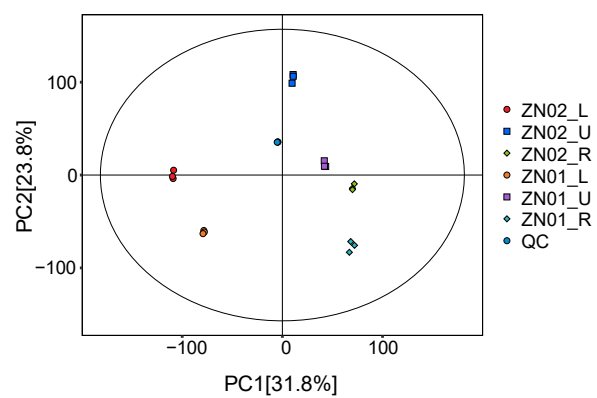

Supplement: Supplementary file 1 [file ijms-26-01558-s001.zip › Supplementary Materials/Supplementary Figures S1-S10/Fig S1.pdf]

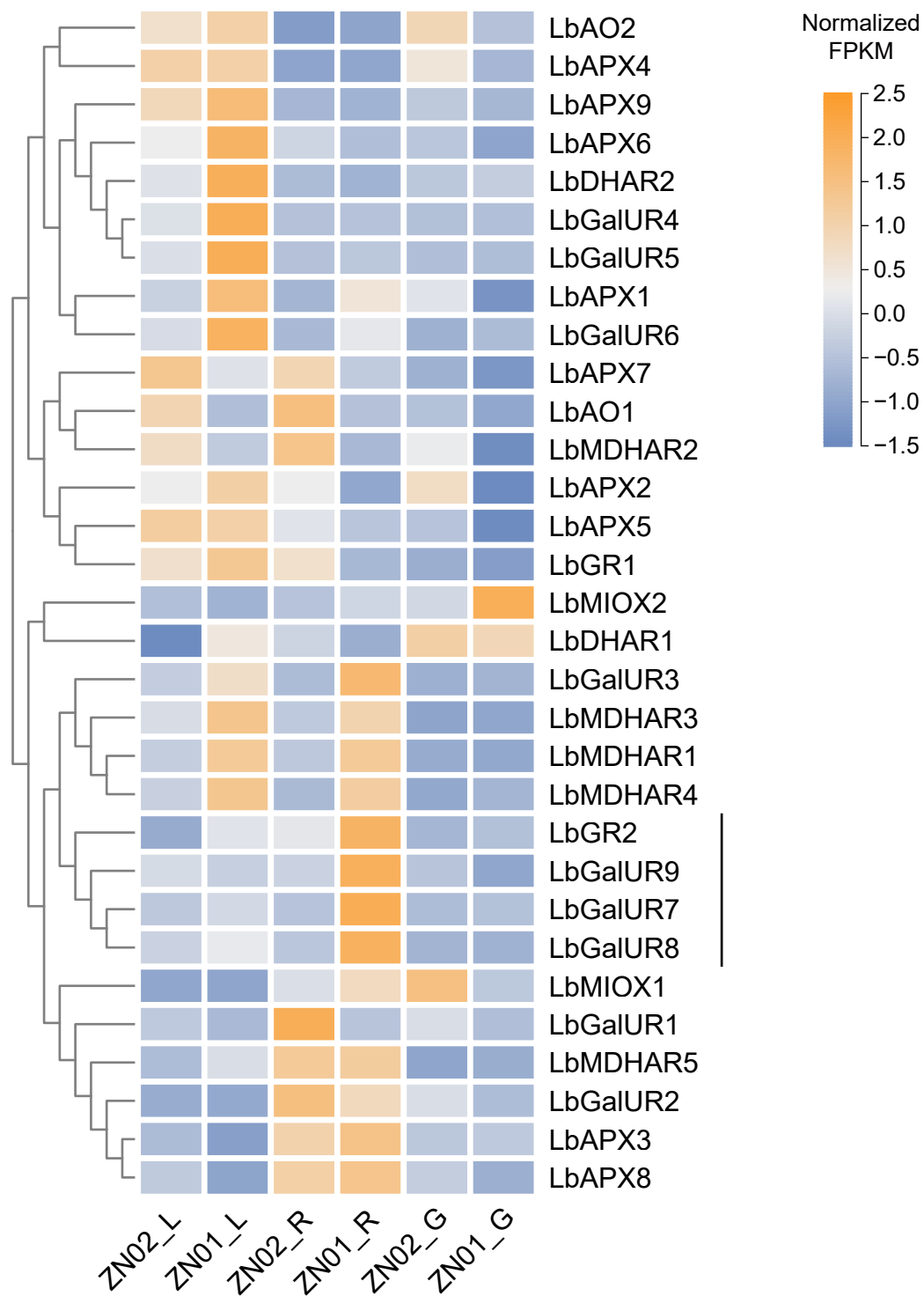

Supplement: Supplementary file 1 [file ijms-26-01558-s001.zip › Supplementary Materials/Supplementary Figures S1-S10/Fig S2.pdf]

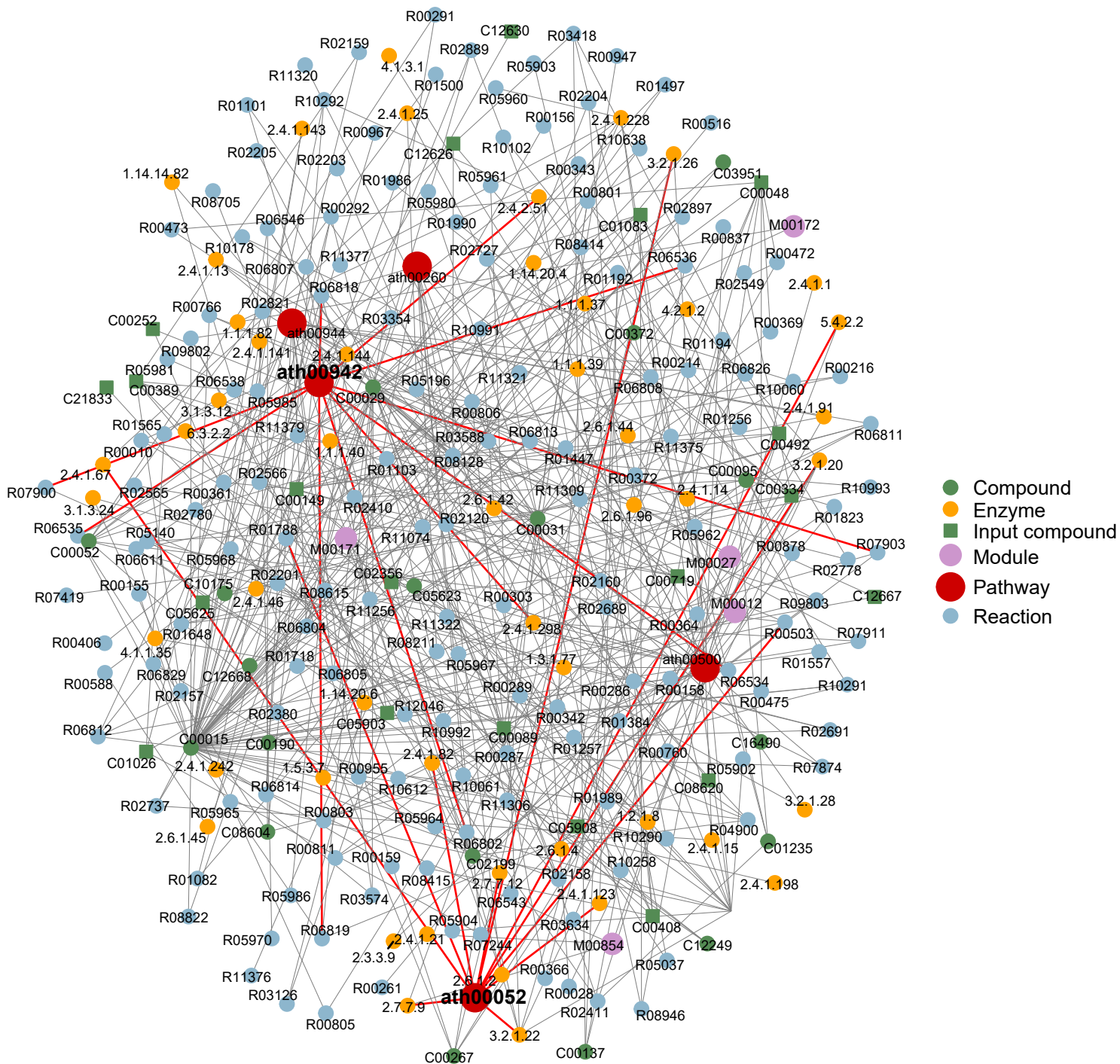

Supplement: Supplementary file 1 [file ijms-26-01558-s001.zip › Supplementary Materials/Supplementary Figures S1-S10/Fig S3.pdf]

A

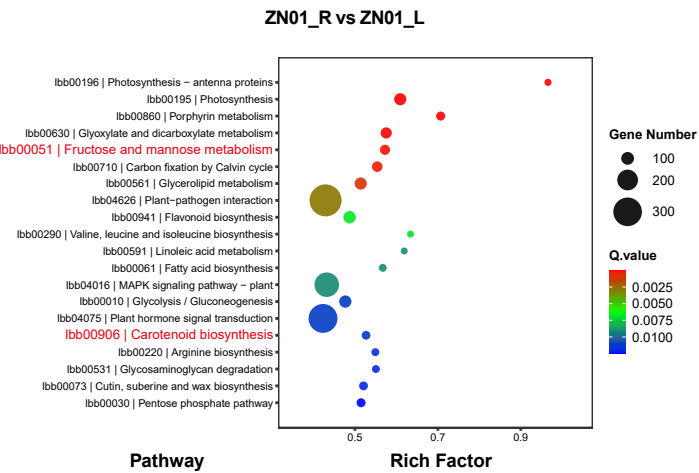

B

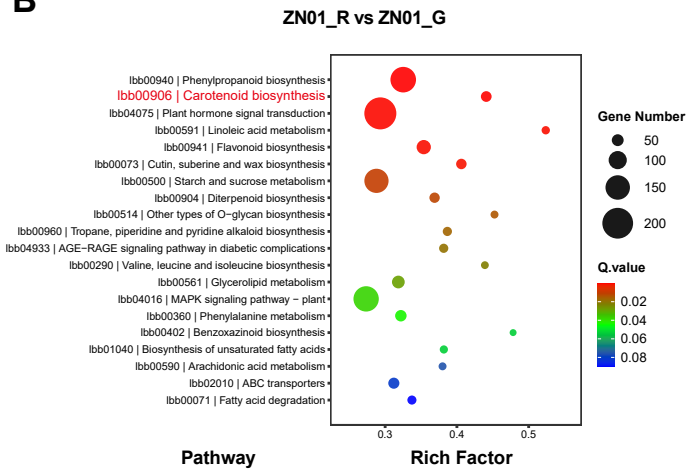

C

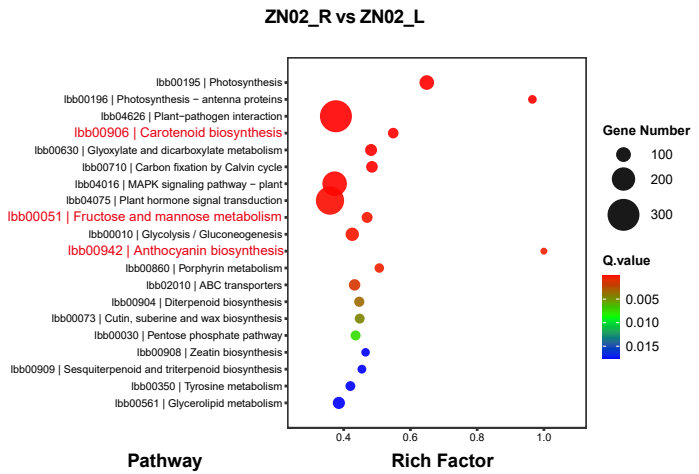

D

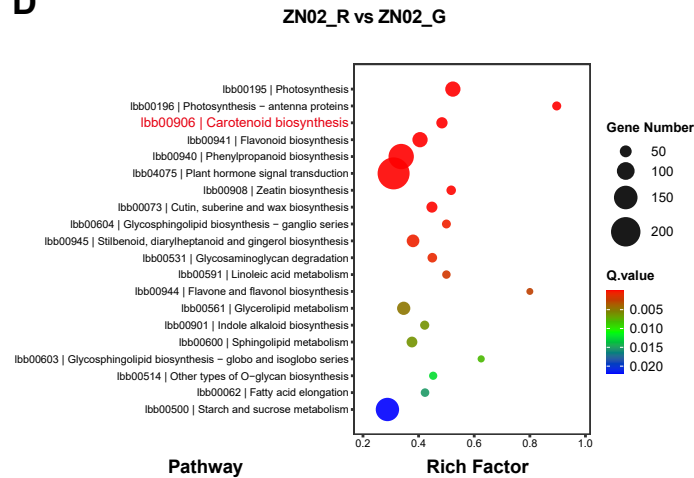

Supplement: Supplementary file 1 [file ijms-26-01558-s001.zip › Supplementary Materials/Supplementary Figures S1-S10/Fig S4.pdf]

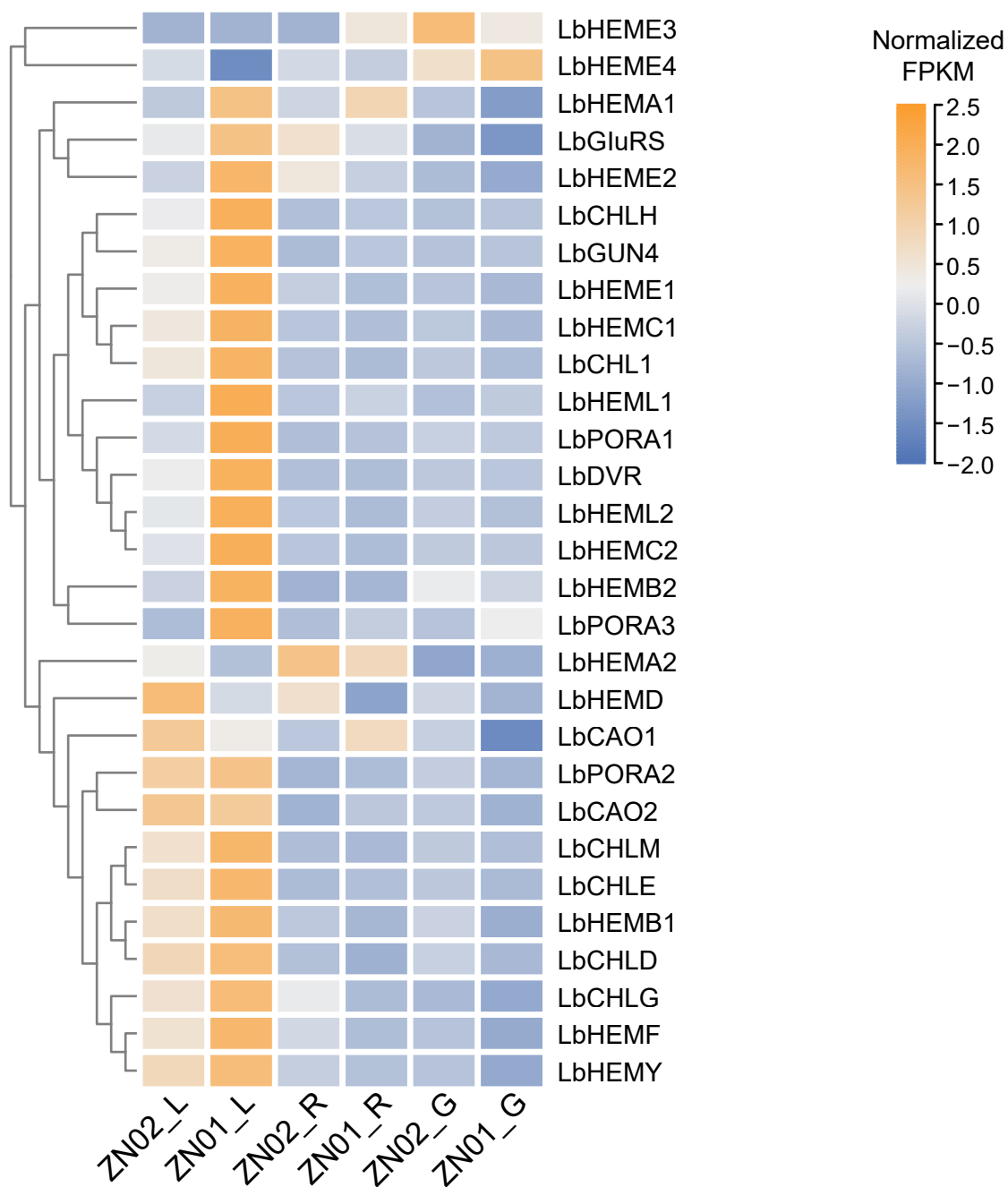

Supplement: Supplementary file 1 [file ijms-26-01558-s001.zip › Supplementary Materials/Supplementary Figures S1-S10/Fig S6.pdf]

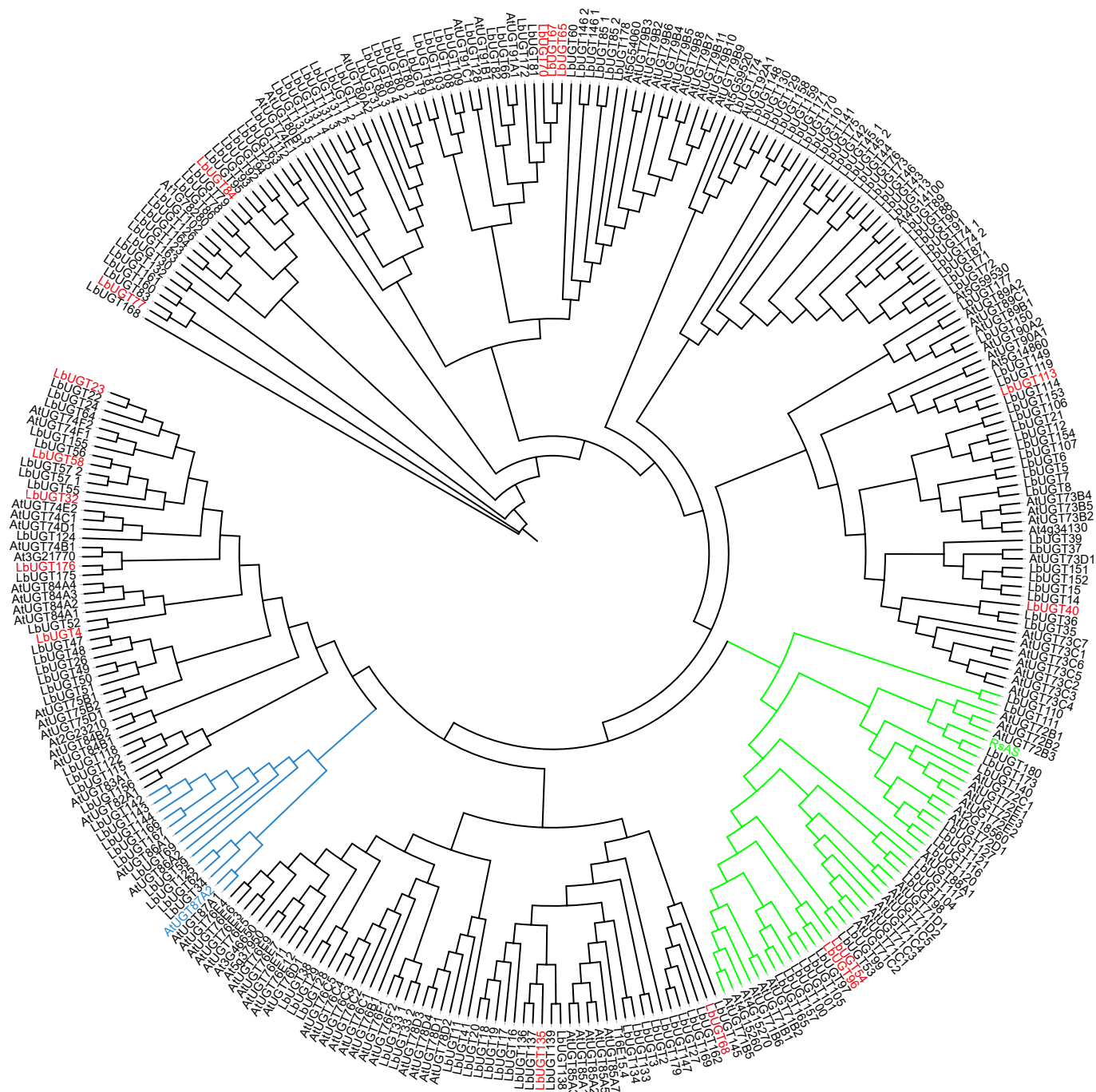

Supplement: Supplementary file 1 [file ijms-26-01558-s001.zip › Supplementary Materials/Supplementary Figures S1-S10/Fig S7.pdf]

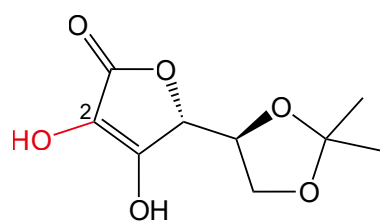

5,6-O-(isopropylidene) ascorbic acid

Galactosidase

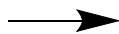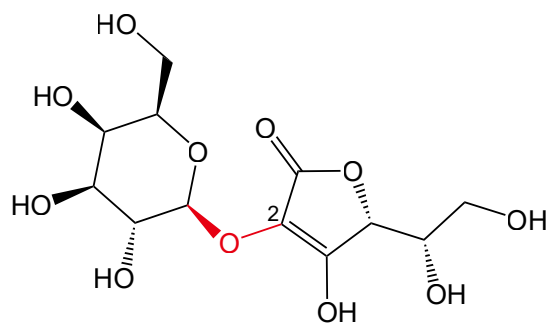

2-O-(β-D-Galactopyranosyl) ascorbic acid

Supplement: Supplementary file 1 [file ijms-26-01558-s001.zip › Supplementary Materials/Supplementary Figures S1-S10/Fig S8.pdf]

**A**

# Cluster Dendrogram

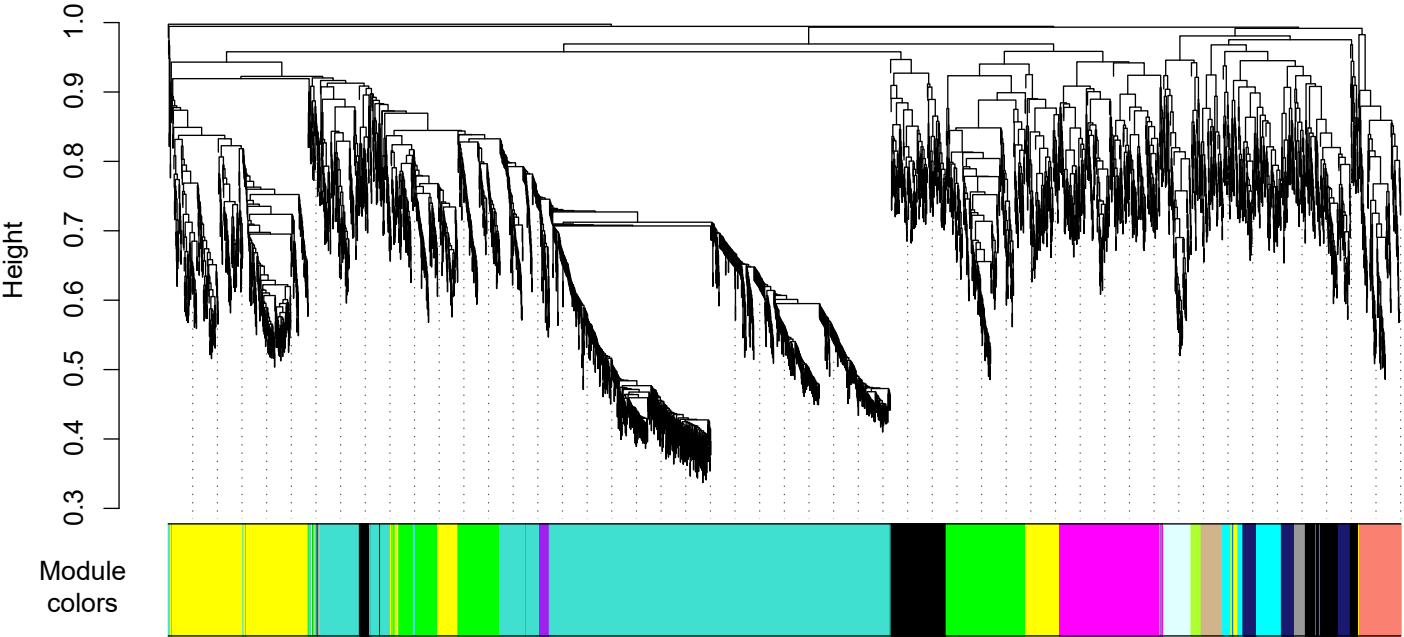

**B**

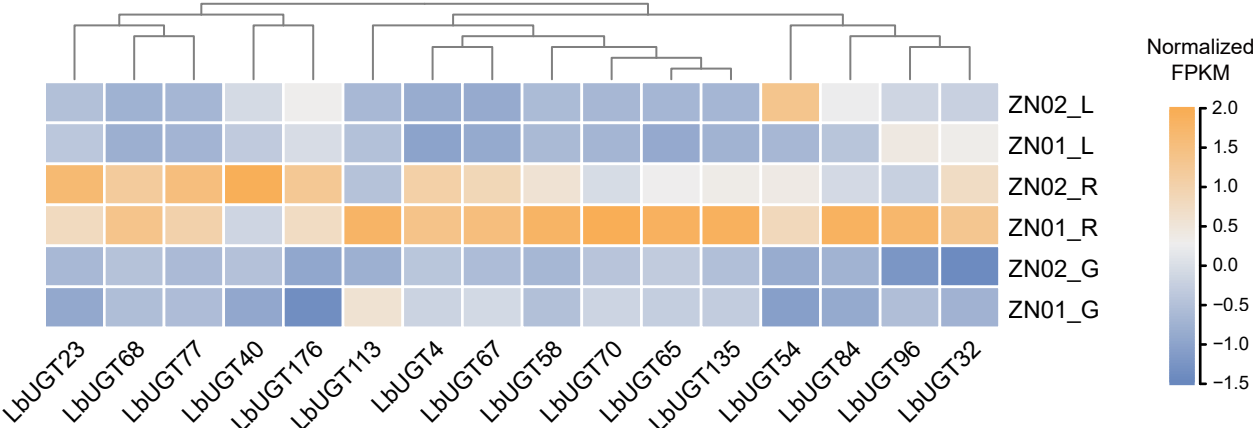

Supplement: Supplementary file 1 [file ijms-26-01558-s001.zip › Supplementary Materials/Supplementary Figures S1-S10/Fig S9.pdf]
